# Novel method for risk stratification of radiation-induced breast fibrosis: subgroup hypothesis verified by machine learning

**DOI:** 10.1038/s41523-026-00980-7

**Published:** 2026-06-12

**Authors:** Ahmad Sami, Petra Seibold, Carsten Sticht, Frank A. Giordano, Marlon R. Veldwijk, Carsten Herskind

**Affiliations:** 1 Cellular and Molecular Radiation Oncology Lab, Dept. of Radiation Oncology, Universitätsmedizin Mannheim, Medical Faculty Mannheim, Heidelberg University, Mannheim, Germany; 2 DKFZ Hector Cancer Institute at University Medicine Mannheim, Heidelberg, Germany; 3 Division of Cancer Epidemiology, German Cancer Research Center (DKFZ), Heidelberg, Germany; 4 Core Facility Platform Mannheim (CFPM), Medical Faculty Mannheim, Heidelberg University, Mannheim, Germany; 5 Department of Radiation Oncology, Universitätsmedizin Mannheim, Medical Faculty Mannheim, Heidelberg University, Mannheim, Germany; 6 Mannheim Institute of Intelligent Systems in Medicine (MIISM), Heidelberg University, Mannheim, Germany

**Keywords:** Biomarkers, Cancer, Computational biology and bioinformatics, Diseases, Oncology, Risk factors

## Abstract

Breast fibrosis (BF) after radiotherapy remains one of the most dreaded late toxicities in breast cancer care, yet multiple additive predictors struggle to capture its underlying biological complexity. Radiation-induced lymphocyte apoptosis (RILA) has recently been associated with the risk of fibrosis more than 10 years post-RT. Here, we show that a combination of five independent factors, RILA, two SNPs in the CTGF and NBS1 genes, and two clinical variables (body-mass index and hypertension) exhibits several important interactions. Partition analysis identified six partly nested subgroups, which could be consolidated into three clinically meaningful risk groups. Machine-learning modelling verified and refined these groups, demonstrating a five-fold variation (17–83%) in BF risk with an AUC = 0.735 in ROC analysis using only these five features. Our study provides proof-of-concept that a biologically realistic subgroup-based approach sharpens predictive performance and may enable clinical identification of a subgroup of breast cancer patients highly susceptible to BF.

## Introduction

Radiotherapy (RT) is an important part of multimodal breast cancer therapy, and with rising numbers of long-term survivors, dose-limiting late reactions in healthy tissues become increasingly important. Thus, for patients treated with breast conserving therapy, subcutaneous fibrosis and telangiectasia can seriously impact tissue function, cosmesis, and patients’ quality of life. In the emerging era of personalised therapy and de-escalation, especially for patients with lower-risk breast cancer, accurate prediction of late toxicity is highly warranted.

Up to 80-90% of the variation in risk between patients has been estimated to be due to non-stochastic factors associated with genetics, clinical characteristics, and lifestyle of the individual patient^1^. Candidate gene studies and genome-wide association studies (GWAS) have led to the notion that multiple single-nucleotide polymorphisms (SNPs) each confer a minor contribution to overall risk in an additive fashion without interactions^2–7^. However, polygenic risk scores (PRS: weighted sums of risks from multiple SNPs) and classical regression models based on dozens or even hundreds of SNPs have not yet delivered clinically useful predictive performance^8–10^.

Radiotherapy-induced side effects in normal tissue are increasingly considered to be caused not by radiation-induced cell death of critical target cells but instead by complex interactions and signaling between different cell types, including immune cells, in a tissue- and endpoint-specific fashion mediated by cytokines and growth factors^11–14^. Since biological processes are controlled through pathways with cross-talk and redundancy, risk factors may not act independently of each other in an additive fashion as described above. Instead, we hypothesise that some risk factors may depend conditionally on other risk factors and be significant only within a particular subgroup of patients. This would dilute the real effect of the risk factor by adding statistical ‘noise’ from complementary subgroups, resulting in inferior p-values when the whole cohort is analysed. Combining ‘omics’ with functional assays might help identify patient subgroups characterised by certain combinations of shared risk factors^15^.

Among predictive functional assays, the radiation-induced lymphocyte apoptosis (RILA) assay on CD8^+^ and/or CD4^+^ T-cell populations (‘killer’ and ‘helper’ T-cells, respectively) is currently the most promising (reviewed in ref. ^15^), showing significant associations with mixed late reactions, breast fibrosis, and telangiectasia^16–19^. However, fibrosis and telangiectasia can manifest several years after radiotherapy, with more than a third of all cases appearing later than five years after treatment^20–22^, making long-term clinical follow-up an essential requirement for stable associations.

The purpose of the present study was to test the subgroup hypothesis by combining the functional CD4^+^ RILA assay at late follow-up (>10 years)^19^ with genomic and clinical risk factors. Two SNPs and two clinical factors were identified as independent features for breast fibrosis and showed interactions with RILA. The decision tree (DT) from predictive partition analysis (PA) yielded six partly nested subgroups, which could be aggregated into three risk groups of high, medium, and low risk of fibrosis. Advanced machine learning (ML) techniques (e.g., ensemble models, deep learning) provide a high degree of stability to the results by incorporating repeated cross validations and have been previously used to develop predictive models based on different combinations of omics, functional, and clinical variables^10,23–26^. Independent random forest (RF) and support vector machine (SVM) showed robust predictions with different characteristics, and combining the two ML models was able to recapitulate the PA risk groups and verify the subgroup hypothesis. This proof-of-concept study suggests that the subgroup hypothesis can identify conditional risk factors that go undetected in classical association studies and may enable efficient risk stratification of patients.

## Results

### Feature selection and test of independence

**Fig. 1:**
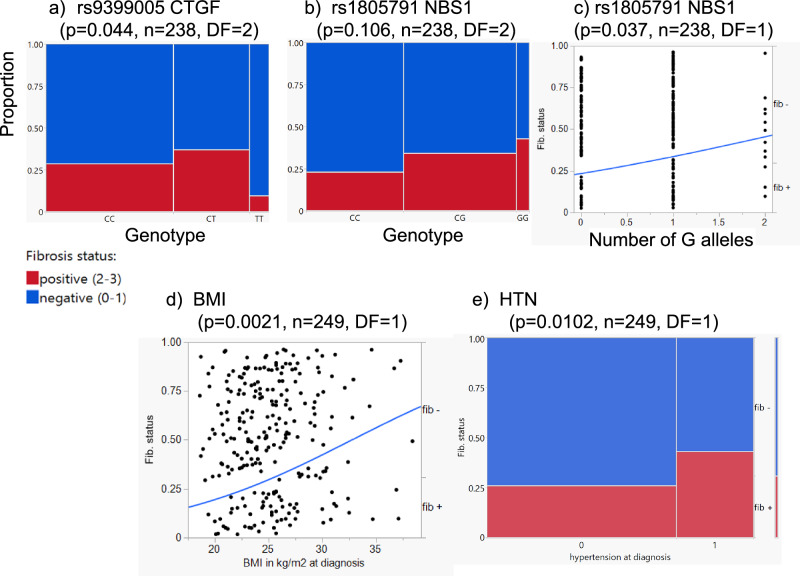
Association between features and radiation-induced fibrosis: grade 2–3 (fib-pos) and grade 0–1 (fib-neg). **a** *CTGF* SNP (Pearson's χ^2^ test); **b** *NBS1* SNP; **c** *NBS1* SNP, number of minor alleles (G), (Bivariate analysis); **d** bodymass index (BMI; logistic regression); **e** Hypertension (HTN; Fisher’s exact test).

Power analyses for the three selected SNPs suggested that OR values in the range 1.77–1.84 could be detected with a power of 0.8 at α = 0.05 in *n* = 238 samples (Supplementary Fig. S1). The association of the SNPs with fibrosis and telangiectasia was tested for each genotype against the two others using Fisher’s exact test (Table 1). The *CTGF* SNP (rs9399005) showed a significant association of the minor allele (TT) with a markedly reduced risk of fibrosis (OR = 0.23, *p* = 0.043; inverse OR = 4.3). This was confirmed in a mosaic plot showing a markedly lower rate of fibrosis in the TT genotype compared with major homozygotes and heterozygotes, which were close to the mean fibrosis rate for the whole cohort (Fig. 1a), suggesting that the minor allele was protective in a recessive fashion.

**Table 1:** The association between late radiotoxicity (breast fibrosis and telangiectasia) and SNP genotypes in the ISE cohort

		ISE cohort (*n* = 238) Fibrosis status			ISE cohort (*n* = 238) Telangiectasia status		
	Geno-type	Fib-pos *n* = 71 pts. (distrib)	Fib-neg *n* = 167 pts. (distrib.)	*p*-value OR (95% CI)	Tel-pos *n* = 20 pts. (distrib)	Tel-neg *n* = 218 pts. (distrib)	*p*-value OR (95% CI)
rs9399005 (CTGF)	CC	39 (54.9%)	97 (58.1%)	0.670 0.88 (0.50–1.54)	9 (45.0%)	127 (58.3%)	0.345 0.59 (0.23–1.47)
	CT	30 (42.3%)	51 (30.5%)	0.10 1.66 (0.94-2.96)	10 (50.0%)	71 (32.6%)	0.140 2.07 (0.82–5.20)
	TT	2 (2.8%)	19 (11.4%)	0.043* 0.23 (0.05–1.00)	1 (5.0%)	20 (9.17%)	1.000 0.52 (0.07–4.10)
rs1805794 (NBS1)	CC	24 (33.8%)	80 (47.9%)	0.047* 0.55 (0.31-0.99)	7 (35.0%)	97 (44.5%)	0.485 0.67 (0.26–1.75)
	CG	41 (57.7%)	79 (47.3%)	0.158 1.52 (0.87–2.27)	11 (55.0%)	109 (50.0%)	0.816 1.22 (0.49–3.07)
	GG	6 (8.4%)	8 (4.8%)	0.365 1.83 (0.61–5.50)	2 (10%)	12 (5.5%)	0.332 1.91 (0.40-9.19)
rs373759 (ATM)	CC	27 (38.0%)	78 (46.7%)	0.254 0.70 (0.40–1.23)	7 (35.0%)	98 (44.9%)	0.483 0.66 (0.25–1.72)
	CT	34 (47.9%)	72 (43.1%)	0.569 1.21 (0.69–2.12)	10 (50.0%)	96 (44.0%)	0.644 1.27 (0.51–3.18)
	TT	10 (14.1%)	17 (10.2%)	0.380 1.45 (0.63–3.37)	3 (15.0%)	24 (11.0%)	0.482 0.43 (0.39–5.23)

p-values (Fisher’s Exact Test, 2-tailed) for each genotype against the other two. *P*-values and odds ratios (OR) with confidence intervals (CI) are shown. An asterisk indicates *p* < 0.05.

The *NBS1* SNP (rs1805794) major homozygote (CC) genotype showed a significant association with lower risk of fibrosis (OR = 0.55, *p* = 0.047; inverse OR = 1.82; Table 1). The rate of fibrosis showed a non-significant increasing trend (*p* = 0.106, Pearson χ^2^ test) from CC to the CG and GG genotypes (Fig. 1b) which was significant when analysed by logistic regression as a function of the number of minor (G) alleles (*p* = 0.037; Fig. 1c). However, because of the small GG group size, minor homozygotes were not analysed separately but were included together with heterozygotes into a single group (CG&GG) associated with increased risk in predictive modelling. No significant association between rs373759 (*ATM*) and fibrosis was detected (Table 1 and Supplementary Fig. S2) nor between any of the three candidate SNPs and telangiectasia (Table 1).

Univariate analysis of clinical features showed significant associations with fibrosis for body mass index (BMI) and hypertension (HTN) (*p* = 0.0028, non-parametric Mann-Whitney U test, and 0.010, Pearson’s χ^2^ test, respectively, *n* = 249; Table 2; Fig. 1d, e). No significant association was observed between the other treatment-related parameters (boost dose, total dose, endocrine therapy, etc.) and fibrosis. Although HTN was significantly associated with higher BMI score (*p* < 0.0001, *n* = 248, logistic regression), multivariate analysis confirmed that both features were individually associated with fibrosis (BMI *p* = 0.016; HTN *p* = 0.055; BMI×HTN *p* = 0.31, *n* = 248). In addition, univariate association analyses were performed in each of 2000 random train/test splits (70/30 splits, keeping the same fibrosis rate in each train/test subset). In each split, the Mann–Whitney U test (BMI) or Fisher’s exact test (HTN) was performed in the training subset (feature “selection”), and the direction/effect was then evaluated in the independent held-out test subset. BMI was significant in 88.6% of training subsets and showed a consistent positive effect direction (fibrosis positive – fibrosis negative > 0) in 98.6% of held-out test subsets (Supplementary Figs. S3a, S3b). Hypertension was significant in 66.5% of training subsets and showed OR > 1/positive risk difference in 96.2% of held-out test subsets (S3c, S3d). As expected for the smaller sample size of the test subsets, statistical significance was less frequent, but the effect directions were highly stable, supporting that these associations are not artefacts of full-dataset selection. This analysis ensures that the effects of selected BMI and HTN features are consistent and coherent across different subsets and were not an artefact of full-dataset selection. Notably, no significant correlation with patient age at treatment was found (*p* = 0.181, *n* = 252), although the rate seemed lower in a small group of younger patients (1/16 patients in the age group 39–46 years), but this was not significant in Pearson’s χ^2^ test of fibrosis versus 5-year age groups (*p* = 0.20, *n* = 252, DF = 7). For telangiectasia, in addition to CD4^+^ RILA, BMI, and pack-years showed significant associations, but as none of the SNPs were significant, telangiectasia was not included in further analysis.

**Table 2:** The association between clinical parameters and fibrosis and telangiectasia status with p-values from the Mann-Whitney U test

Clinical parameter	Fibrosis negative	Fibrosis positive	*p*-value	Telangiectasia negative	Telangiectasia positive	*p*-value
Age at treatment (median)	*n* = 175 59	*n* = 77 60	0.181	*n* = 228 59	*n* = 24	0.23
BMI in kg/m^2^ at diagn. (median)	*n* = 172 24.25	*n* = 77 25.70	0.003*	*n* = 225 24.8	*n* = 24 26.15	0.041*
BH cup size (median)	*n* = 162 B	*n* = 76 C	0.169	*n* = 215 B	*n* = 23 C	0.32
Smoking status current past never	*n* = 168 28 (16.7%) 27 (16.1%) 113 (67.3%)	*n* = 77 11 (14.3%) 16 (20.8%) 50 (64.9%)	0.65	*n* = 221 34 (15.4%) 36 (16.3%) 151 (68.3%)	*n* = 24 5 (20.8%) 7 (29.2%) 12 (50.0%)	0.170
Smoking Pack-years (75% quantile)	*n* = 164 2.4	*n* = 76 12.6	0.29	*n* = 216 3.7	*n* = 24 22.2	0.020*
Allergy at diagnosis	46/173 (26.6%)	23/77 (29.9%)	0.64	62/226 (27.4%)	7/24 (29.1%)	0.86
Hypertension at diagnosis	41/172 (23.8%)	31/77 (40.3%)	0.007*	6/225 (27.6%)	10/24 (41.7%)	0.160
Acute side effects	16/61 (20.8%)	34/141 0.46	0.46	44/228 (19.3%)	6/24 (19.8%)	0.59

Some patients did not have complete clinical data. An asterisk indicates *p* < 0.05.

Thus, two SNPs (rs9399005 and rs1805794), two clinical features (BMI and HTN), and CD4^+^ RILA, were available for predictive modelling of breast fibrosis. The independence of these five features with respect to fibrosis was demonstrated by low Variance Inflation Factors, VIF = 1.03–1.10, showing minimal collinearity (Table 3), i.e., none of the features could be explained by the other four.

**Table 3:** Variance Inflation Factor (VIF) analysis of collinearity between CD4^+^ RILA, the two significant SNPs, body mass index (BMI), and hypertension (HTN)

Feature	VIF
CD4^+^ RILA	1.06
CTGF_(TT)	1.03
NBS1_(CC)	1.03
BMI	1.10
HTN	1.07

VIF ∼1 indicates little or no collinearity and VIF > 5 indicates strong collinearity. Thus, none of the five features can be replaced by one of the other features in a regression model.

### Subgroups and interaction effects

**Fig. 2:**
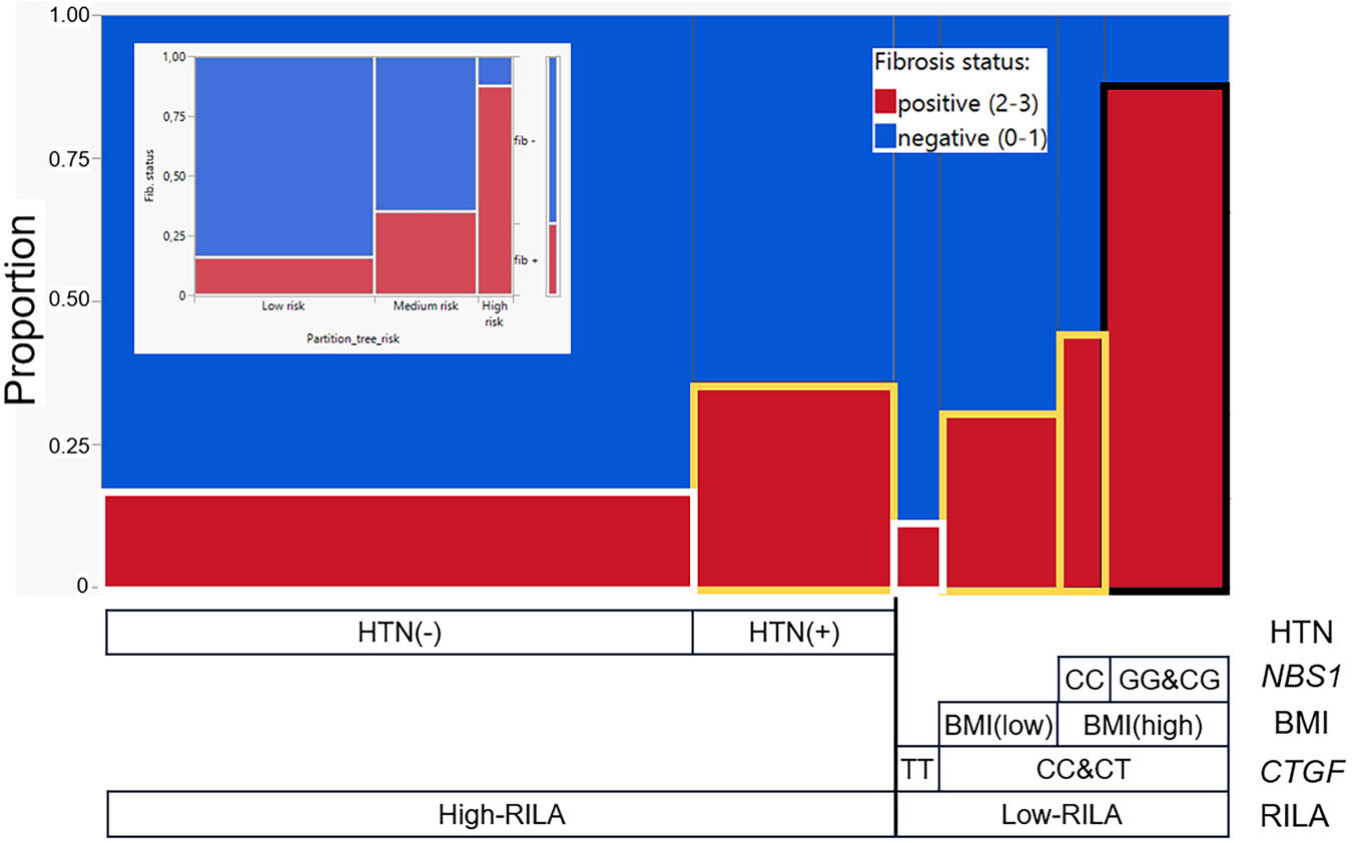
Six subgroups after five splits by partition analysis of *n* = 219 patients with complete data for the five features: CD4^+^ RILA, *CTGF* and *NBS1* SNPs, body-mass index (BMI), and hypertension (HTN). Distribution of fibrosis-positive (grade 2–3) and negative (grade 0–1) samples after PA analysis. High-RILA: ≥7.48%, low-RILA: <7.48%; *CTGF* TT vs CC&CT; *NBS1* CC vs CG&GG; BMI(high): ≥24.4 kg/m^2^, BMI(low): <24.4 kg/m^2^, HTN(+): hypertension, HTN(-): normal blood pressure. Insert shows the three risk groups by aggregating the two low-risk groups and three medium-risk groups, respectively.

In order to identify potential subgroups of patients with common risks, predictive PA was performed. Complete case analysis was performed (n = 219), excluding fifteen samples with missing CD4^+^ RILA values and four with missing BMI and/or hypertension data. The overall rate of fibrosis was 30.1%, and the rates in the six subgroups resulting from the first five splits are shown in Fig. 2 and the corresponding DT, including the number of patients in each subgroup, in Supplementary Fig. S4. The subgroup characterised by low-RILA, non-TT *CTGF* genotype, BMI(high) plus CG&GG *NBS1* genotype showed a significantly increased rate of fibrosis (87.5%, *p* < 0.0001; relative risk = 3.79 [2.81; 5.11]), whereas the rate was significantly reduced (16.5%, *p* < 0.0001; relative risk = 0.37 [0.23; 0.58]) in high-RILA patients without HTN.

Contingency analyses for the low-RILA and high-RILA subgroups showed significant effects of the two SNPs and BMI in the low-RILA subgroup but not in the complementary high-RILA subgroups, whereas the opposite was true for HTN (Supplementary Fig. S5). In the case of the *CTGF* SNP, the TT genotype was equally protective in both subgroups, and the absence of significance in the high-RILA subgroup was due to the lower background risk. Subdivision of RILA subgroups according to BMI status showed that the *NBS1* SNP was a significant risk factor (*p* = 0.009) only in the low-RILA + BMI(high) subgroup but not in the other three subgroups (*p* = 0.52–1.00), consistent with this risk factor being conditional on RILA and BMI (Supplementary Fig. S6).

Interaction tests of genomic and clinical features with CD4^+^ RILA showed significant interactions with RILA for the effects of rs1805794, BMI, and HTN, but not rs9399005 (Table 4). The influence of the SNPs on CD4^+^ RILA showed a recessive effect of the T allele with lower RILA values in the *CTGF* TT than the CC and CT genotypes (Supplementary Fig. S7a). Since low RILA values were associated with increased risk of fibrosis, whereas TT was protective, this implies that the protective effect dominated over the effect of the RILA values. The *NBS1* genotypes were consistent with a dosage effect of the minor allele (Supplementary Fig. S7b), but no significant association was found for the *ATM* SNP (Supplementary Fig. S7c). Cumulative distribution functions (CDF) for the *CTGF* and *NBS1* SNPs corroborated the recessive and additive effects, respectively (Supplementary Fig. S7d,e). The correlation between RILA and BMI was very weak (r^2^ = 0.02, *p* = 0.02, *n* = 234, linear regression) while none was observed for HTN (*p* = 0.18, *n* = 234, Mann-Whitney) (Supplementary Fig. 7f,g).

**Table 4:** Test of interactions between CD4^+^ RILA and SNPs or clinical features, respectively, for the risk of fibrosis corresponding to the partition analysis (PA) decision tree (DT)

Interactions	Coeff	OR	OR_CI low	OR_CI high	p-value	p_FDR adj.
RILA:CTGF(TT)	0.0352	1.036	0.913	1.175	0.59	0.59
RILA:NBS1(CC)	−0.905	0.405	0.190	0.862	0.019	0.029*
RILA:BMI	−0.0264	0.974	0.957	0.991	0.0026	0.0154*
RILA:HTN	0.1572	1.170	1.048	1.307	0.0052	0.0157*
NBS1(CC):BMI	0.261	0.770	0.513	1.156	0.21	0.25
RILA: NBS1(CC):BMI	0.0386	1.039	1.008	1.072	0.013	0.026*

Significant interactions were found between RILA and NBS1(CC), BMI, and HTN, respectively, but not between RILA and CTGF(TT). BMI and NBS1(CC) showed no significant interaction, whereas the interaction between RILA, NBS1(CC), and BMI was significant. An asterisk indicates p_FDR adj.<0.05.

**Fig. 3:**
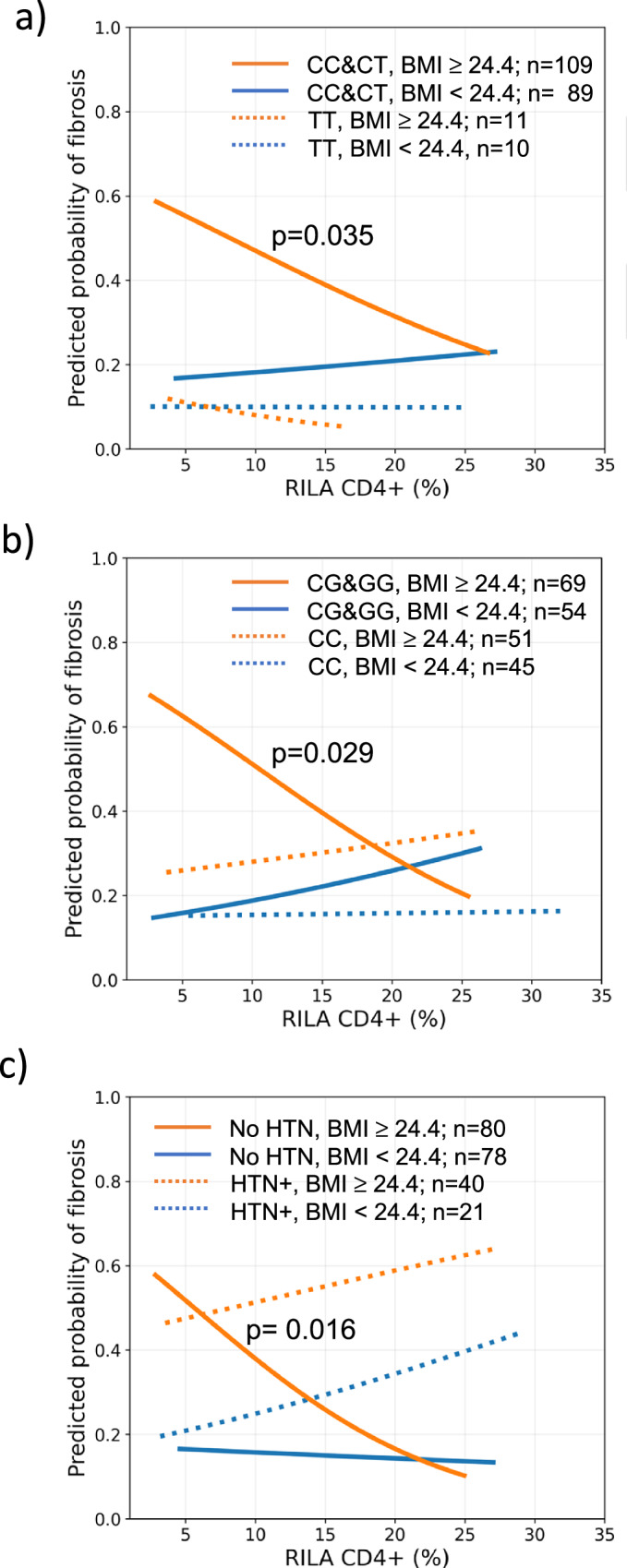
Feature interactions showing risk of fibrosis (grade 2–3) as a function of CD4^+^ RILA according to BMI subgroup, BMI(high)≥24.4 kg/m^2^, BMI(low)<24.4 kg/m^2^. **a** *CTGF* SNP; **b** *NBS1* SNP; **c** HTN. Logistic regression was based on all data within each group, but the fitted curves show only the ranges corresponding to 95% of the data (excluding outliers).

Because BMI itself showed a trend (p = 0.11) for an association with fibrosis even in the high-RILA subgroup (Supplementary Fig. S5e), the interaction effects were analysed in more detail by logistic regression of the risk of fibrosis versus RILA for each of the three features, rs9399005, rs1805794, and HTN, split according to BMI(high) and BMI(low) (Fig. 3a–c and Supplementary Table S1a). This confirmed that RILA had no influence on fibrosis in the TT *CTGF* genotype (*p* = 0.35 and *p* = 0.98, respectively), whereas the association of fibrosis risk with low RILA was strong in the CC&CT genotype with BMI(high) but not BMI(low) (p = 0.035 and p = 0.61, respectively). A similar dependency on RILA was observed for BMI(high) patients with *NBS1* CG&GG genotypes (p = 0.029) but not for the three other combinations (*p* = 0.32–0.96). Increased fibrosis at low RILA values was also seen in BMI(high) but not BMI(low) patients with normal blood pressure (*p* = 0.016 and *p* = 0.79, respectively). An apparently inverted relationship of fibrosis risk with *higher* RILA values in HTN+ patients was not significant in logistic regression for BMI(high) and BMI(low) (p = 0.46 and p = 0.23), respectively, although the fibrosis levels were significantly different (*p* = 0.03). Average marginal effect analysis reduced the p-values slightly, confirming the significant as well as the non-significant p-values except for HTN+ patients with BMI(low) where the *p*-value for RILA dependence decreased to reach significance (*p* = 0.024) (Supplementary Table S1b). Thus the association between increased fibrosis and low RILA values was observed in non-TT *CTGF* genotypes with CG&GG *NBS1* genotype, BMI(high), and no hypertension, and was indeed only significant in the subgroup having all three characteristics (*p* = 0.015, *n* = 44, and *p* = 0.80, *n* = 154 respectively).

By aggregating the two subgroups with low risk and three subgroups with medium risk, patients could be assigned to three risk groups (high, medium, low) with a highly significant (*p* < 0.0001) five-fold difference in risk (Table 5 and insert in Fig. 2).

**Table 5:** Risk groups for breast fibrosis based on aggregating subgroups from the partition analysis (PA) decision tree (DT)

Risk group	Subgroups	No. of patients (proportion)	Fibrosis positive (risk)	OR (95% CI)
Low	**(1)** RILA ≥ 7.48% & normal blood pressure (no HTN) **(3)** RILA < 7.48% & *CTGF*(TT)	124 (56.6%)	20 (16.1%)	0.20 (0.11–0.38)
Medium	**(2)** RILA ≥ 7.48% & HTN **(4)** RILA < 7.48% & *CTGF*(CC&CT) & BMI < 24.4 kg/m² **(5)** RILA < 7.48% & *CTGF*(CC&CT) & BMI ≥ 24.4 kg/m² & *NBS1*(CC)	71 (32.4%)	25 (35.2%)	1.42 (0.77–2.60)
High	**(6)** RILA < 7.48% & *CTGF*(CC&CT) & BMI ≥ 24.4 kg/m² & *NBS1*(CG&GG)	24 (11.0%)	21 (87.5%)	23.3 (6.7–81.8)

*RILA* Percent CD4^+^ radiation-induced lymphocyte apoptosis, *BMI* body mass index, *HTN* hypertension. The subgroup numbers **(1-6)** refer to the order of the subgroups in Fig. 2 from left to right.

### Verification of the subgroup hypothesis by machine learning

**Fig. 4:**
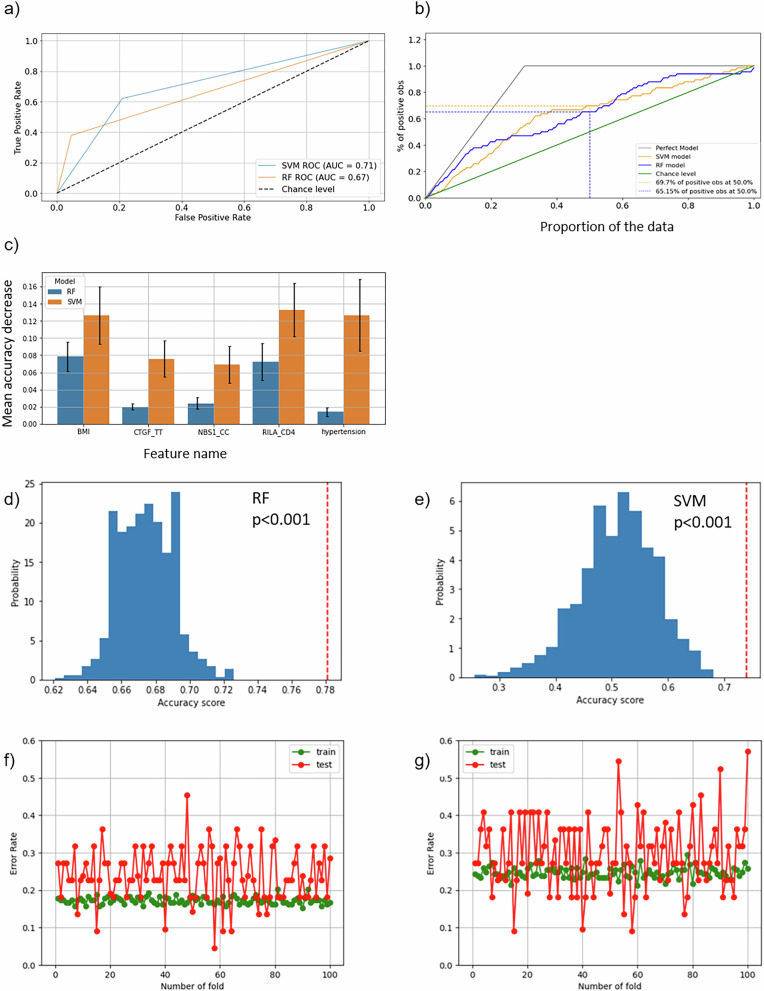
Evaluation of predictive performance and robustness of random forest (RF) and support vector machine (SVM) models. **a** receiver operating characteristic (ROC) analysis; **b** cumulative accuracy profile (CAP) analysis; **c** feature importance; **d** permutation significance test (accuracy score) for RF; **e** permutation significance test (accuracy score) for SVM; **f** 10 times 10-fold cross-validation of RF; **g** 10 times 10-fold cross-validation of SVM.

Because the partition model carries a risk of overfitting the data, robust predictive ML models (RF and SVM) were developed as an independent test of the subgroup hypothesis. The RF model was more precise in predicting fibrosis positive samples (PPV of 78% versus 56% for SVM), while SVM was marginally more precise in predicting fibrosis negative patients (NPV of 83% versus 78% for RF) (Supplementary Table S2a). The AUC from ROC analysis was 0.67 for RF and 0.71 for SVM (Fig. 4a). CAP analysis showed rather similar accuracies for the two models (AR = 0.40 and 0.37 for RF and SVM, respectively) but different characteristics, with RF discriminating more extreme samples (high and low probabilities for fibrosis) and SVM performing better in the middle range (Fig. 4b). Brier scores were computed (RF = 0.183, SVM = 0.188) and the calibration curves generated based on the predicted probability values (Supplementary Fig. S8). The predictions for the RF and SVM models are presented as a confusion matrix showing true and false positive and negative predictions (Supplementary Table S2b).

Analysing the importance of the five features confirmed CD4^+^ RILA and BMI as the two most important features in both models, while the two SNPs were less important, especially in RF (Fig. 4c). Hypertension showed high importance in SVM but little importance in RF. Notably, the decrease in accuracy was lower for every feature in RF, indicating greater robustness.

A permutation significance test showed both models to be statistically highly significant with very low probability that the accuracy values were a chance result (RF: *p* < 0.001, SVM: *p* < 0.001) (Fig. 4d,e). Stratified 10-fold cross validation showed mean error rates of 0.17 ± 0.01 (s.d.) for the training data set and 0.24 ± 0.07 in the test set for the RF model (Fig. 4f), and 0.25 ± 0.015 and 0.30 ± 0.09, respectively, for the SVM model (Fig. 4g). Thus, although the RF model was slightly more stable than SVM, the difference in accuracy between training and test datasets of less than 0.1 in both models confirmed their robustness.

**Fig. 5:**
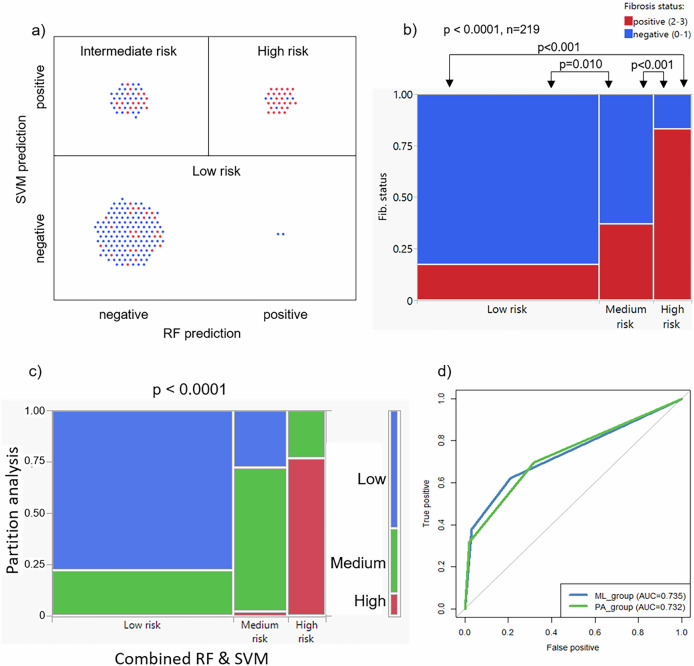
Classification of patients into three risk groups based on combined machine learning (ML) models: random forest (RF) and support vector machine (SVM). **a** Normal Mixture Clustering of prediction by SVM versus RF. **b** association between combined ML risk groups and fibrosis status; **c** correlation between partition analysis (PA)-derived and combined ML risk groups; **d** receiver operating characteristic (ROC) analysis of the PA and combined ML risk group models. AUC area under the curve.

A comparison of predictions by the two models (Fig. 5a) showed that SVM discriminated a low-risk group (n = 146, 66.7%) with 17.1% fibrosis (i.e., false negatives). The remaining 33.3% of the patients were divided into a high-risk group predicted to be fibrosis-positive by both models (*n* = 30, 13.7%) with 83.3% fibrosis and a group predicted to be fibrosis-positive by SVM but fibrosis-negative by RF (*n* = 43, 19.6%) showing 37.2% fibrosis (i.e., medium risk). Considering these observations, the two models were combined to stratify the risk of fibrosis as follows: low-risk group (predicted negative by SVM), medium-risk group (predicted positive by SVM and negative by RF), and high-risk group (predicted positive by both SVM and RF) (Table 6). Thus, the combined ML model (RF + SVM) defined three risk groups spanning a 4.9-fold difference in risk of fibrosis (Fig. 5b), which were highly correlated with the risk groups defined by PA (*p* < 0.0001) (Fig. 5c). Notably, none of the patients were classified as low risk by one method and high risk by the other method, and no patient with *CTGF* TT genotype was found in the ML high-risk group. The combined ML model verified 23/24 (95.8%) of PA high-risk patients, representing a well-defined subgroup characterised by *NBS1* CG&GG in combination with low-RILA and BMI(high), excluding *CTGF* TT genotypes. In addition, the combined ML model assigned seven patients from the PA medium-risk group to the high-risk group, of which five (71%) were fibrosis-positive. Four of the seven were characterised by *high* RILA values (22.3-29.7%), heterozygous *NBS1* genotype (CG), moderate overweight (BMI = 25.2–25.5 kg/m^2^) to obesity (BMI = 30.9 kg/m^2^), and HTN, while the remaining three had *low* RILA values (3.8–7.0%), *NBS1* major homozygous genotype (CC), and were obese (BMI = 30.0–32.5 kg/m^2^) without hypertension. Thus, combinations of certain high-risk factors seem to compensate for the absence of one of the high-risk factors, low-RILA or *NBS1* CG&GG genotype. However, these unplanned observations on small numbers of patients are exploratory, and further studies on larger cohorts are needed to confirm if the combined ML model is indeed able to weigh risk factors against each other.

**Table 6:** Risk groups for breast fibrosis based on the combined machine learning model (RF + SVM)

Risk group	RF predicted	SVM predicted	No. of patients (proportion)	Fibrosis positive (risk)	OR (95% CI)
Low	Negative or Positive	Negative	146 (66.7%)	25 (17.1%)	0.16 (0.09–0.30)
Medium	Negative	Positive	43 (19.6%)	16 (37.2%)	1.49 (0.74–3.01)
High	Positive	Positive	30 (13.7%)	25 (83.3%)	18.0 (6.5–50.1)

Odds ratios (OR) and 95% confidence intervals (CI) were calculated for the likelihood of fibrosis in each risk group against the rest of the cohort.

The combined ML model also verified 111/134 patients (82.8%) from the PA low-risk group (high-RILA, no HTN or low-RILA, TT *CTGF* genotype). The ML medium-risk group was smaller than the PA medium-risk group (19.6% versus 32.4% of the patients), and included only a single patient classified as high-risk by PA. AUC values from ROC analysis of the three risk groups were similar for both models (0.735 and 0.732 for combined ML and PA, respectively), but the combined ML model discriminated more high- and low-risk patients with only marginally increased errors (Fig. 5d; compare Tables 5 and 6). Thus, the combined ML model was able to assign 80.4% of the patients to low- or high-risk groups with 83% accuracy (NPV = 0.829 and PPV = 0.833), respectively, i.e., with approximately 17% overall error.

The performance of the RF and SVM models was clearly superior to LR (as a representative for classical predictive models) regarding PPV, recall Fib-, accuracy, kappa coefficient, and AUC. Furthermore, they also showed better kappa and AUC values than other common ML models, XGB, KNN, and NBC (Supplementary Table S2a). Importantly, the AUC for the combined ML model was markedly higher than for LR or any of the individual ML models.

### Functional effect of the rs9399005 (*CTGF*) TT genotype

**Fig. 6:**
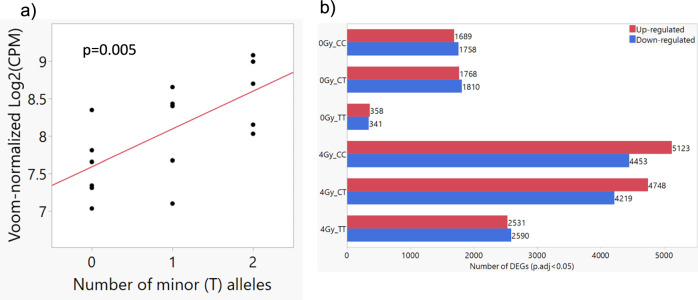
Functional test of the *CTGF* SNP. **a** Increased expression levels of *CTGF* mRNA (log2(CPM) as a function of the number of rs9399005 minor alleles (*p* = 0.005). **b** The number of up- and down-regulated genes (p.adj < 0.05) in fibroblasts with different genotypes on day 2 after irradiation with 4 Gy or sham (0 Gy) relative to untreated cells on day 0.

Because of its position in the non-coding 3’ region sequence, we speculated that the protective *CTGF* TT genotype (rs9399005) might affect *CTGF* gene expression or downstream genes regulated by CTGF. Untreated cultures of six fibroblast strains of each genotype (one TT strain was excluded from the analysis because of poor growth) showed an up to a two-fold increase of basal *CTGF* mRNA levels (day 0) with increasing number of T-alleles (Fig. 6a). Incubation for 2 days after irradiation with 4 Gy increased the number of differentially expressed genes (DEGs) more than in sham-irradiated controls (0 Gy). The number of up- and downregulated DEGs in TT genotypes was significantly lower compared with the CC or CT genotypes (Fig. 6b; Supplementary Data 1: for legend, see Supplementary Material). This may be related to the slower proliferation of TT fibroblasts, which remained subconfluent while CC fibroblasts reached confluence after two days of incubation (not shown). These results show that the TT genotype influences fibroblasts’ phenotype at the transcriptional level.

## Discussion

In this study, we tested the subgroup hypothesis that small-to-modest effect sizes of SNPs on adverse late reactions may result from some risk factors being conditional on certain other risk factors^15^. By combining the functional RILA assay with two genomic and two clinical features, the PA DT identified six partly nested subgroups. The two SNPs and BMI had significantly larger effects in the low-RILA than in the high-RILA subgroup, whereas hypertension was significant in the high-RILA but not the low-RILA subgroup. In particular, the NBS1 CG&GG genotype was conditional on low-RILA and BMI(high). By contrast, the CTGF TT genotype was protective in both RILA subgroups and dominated over the increased risk associated with low CD4^+^ RILA.

Interaction analysis confirmed that the *NBS1* SNP and both clinical features interacted with CD4^+^ RILA and showed that the association between low-RILA and fibrosis was confined to patients with the combination of non-TT *CTGF* genotype, CG&GG *NBS1* genotype, high BMI, and no hypertension. The six subgroups were aggregated to produce three PA risk groups with five-fold variation in risk, which were verified by robust ML modelling. In particular, 23/24 patients (96%) in the unaggregated PA high-risk group were also identified as high-risk by the combined ML model, demonstrating that this PA subgroup was not a result of overfitting. This provided proof-of-concept that the increased risk associated with the *NBS1* G-allele is conditional on other risk factors and that its significance is obscured when analysing the whole cohort. To the best of our knowledge, the present study is the first to show an association of this SNP with fibrosis.

The combined ML model incorporated the complex feature interactions by weighing certain risk factors against the combination of other risk factors. The different CAP characteristics of the RF and SVM models can be attributed to differences in the way they handle sample classification. The RF model classifies samples that align with the core characteristics of their respective opposite class, whereas SVM maximises the decision boundary and excels in correctly identifying samples such as inner outliers sharing characteristics with the opposite class. Importantly, both RF and SVM individually outperformed the classical approach to predictive modelling represented by LR.

The assignment of seven PA medium-risk patients to the ML high-risk group indicates that, in some cases, other risk-factor combinations or a higher grade of BMI may compensate for the absence of low-RILA or *NBS1*(CG&GG). Furthermore, the recessive protection by the CTGF T-allele did not rely on other risk factors. Thus, subgroups may be formed in different ways, probably depending on the role of risk factors in mechanistic pathways.

The combined ML model was able to correctly predict 25/30 (83%) fibrosis-positive patients with fibrosis in the high-risk group (13.7% of the cohort) and 121/146 (83%) fibrosis-negative patients in the low-risk group (67% of the cohort), representing a five-fold difference in risk levels (83% and 17.1%, respectively). In a clinical setting, this would potentially enable precise risk stratification by assigning 80% of the patients to the high- and low-risk groups with 17% errors. The identification of a minor group of patients (∼1 in 7) with a 2.8-fold increased risk would allow clinicians to offer high-risk patients an alternative, normal-tissue sparing treatment such as partial-breast radiotherapy with the potential to reduce the overall rate of fibrosis by more than a third. However, the model needs to be validated in an independent cohort before it can be applied routinely outside clinical studies.

Although the patients were accrued prospectively, this was an observational study with a moderate sample size, and blood samples for the RILA assay were obtained at the late follow-up examination. However, CD4^+^ lymphocytes are not part of standard RILA protocols and, as far as is known, no cohort with comparable long-term follow-up is available, so a new study will be needed for external validation before clinical application.

The AUC was similar for the PA and ML approaches, but the more robust, combined ML model performed marginally better than PA with respect to assigning more fibrosis-positive patients to the high-risk group and more fibrosis-negative patients to the low-risk group, with minimal losses of accuracy while decreasing the size of the medium-risk group. This resulted in a smaller medium-risk group and additional patients assigned to the high- and low-risk groups, suggesting that the more robust, combined ML model may weigh high values of some risk factors against the absence of others. However, these explorative observations on small numbers of patients need to be confirmed in a large study. To the best of our knowledge, no other predictive model has identified a subgroup of high-risk patients with a 2.8-fold increased risk (83.3%) compared with the whole cohort (30.1%).

It is important to note that the PA cutpoints for CD4^+^ RILA and BMI were not used in ML modelling, which used continuous values. However, stochastic fluctuations in distributions of features between different cohorts could potentially affect the sensitivity of the ML models. To address this issue, stratified 10-fold cross-validation was performed, indicating overall solid reproducibility, but marginally higher stability of the RF compared to the SVM model. Nevertheless, the generalisability of the model and the quantitative risk of fibrosis in the risk groups need to be confirmed in an independent data set.

PRSs comprising a few dozen SNPs showed highly significant associations with late reaction in breast cancer patients at two-year follow-up, but data on the predictive performance were not presented^8^. LR was used in an early breast cancer cohort incorporating several clinical and genomic features, which reported a misclassification error of 34%^26^. Only a few studies have used ML modelling for predicting late normal tissue reactions after radiotherapy. In a previous study, six different ML models were used to predict radiotherapy outcome and three toxicities in patients treated for different cancers ((non-)small-cell lung cancer, head-and-neck cancer, meningioma)^27^. The models were developed and evaluated on 12 separate datasets, but no best model was found for all datasets. For the RF model, a broad range of AUC values was found with a median of 0.725, i.e., comparable to the simpler and biologically more specific model in the present study.

A pre-conditioned random forest regression (PRFR) with SNPs from a GWAS on prostate cancer patients yielded AUC values of 0.55–0.70 in ROC analysis for four genitourinary toxicities, and optimal performance required at least 75% of several hundreds of SNPs^10^. More recently, the REQUITE cohort has been used to validate candidate SNPs identified from the literature with modest results^25^. A similar approach, selecting sets of 13 interacting SNPs, yielded AUC values of 0.78 for late urinary frequency ≥gr.2 and 0.71 for late haematuria ≥gr.1, but just 0.63–0.68 for three other endpoints^24^. Using only five features, the present model performed on par with much more complex models and has scope for further improvement.

The general question whether a predictive test will indeed widen the therapeutic window may be addressed by a cost-benefit analysis as estimated for effects on normal lung tissue relative to adjusting radiotherapy prescription dose according to genomic markers of non-small cell lung cancer radiosensitivity^28^. In the case of breast radiotherapy, intraoperative radiotherapy (IORT) with 50 kV X-rays results in hardly any fibrosis if given alone without EBRT^29^ but a cost-benefit assessment will need to be included as part of a carefully planned, prospective clinical trial.

Regarding mechanistic aspects, the association of low RILA values with increased risk of fibrosis emphasises that lymphocyte apoptosis is not a surrogate for the radiosensitivity of patients’ normal tissue cells and instead supports an involvement of the immune system in radiation-induced fibrosis^15^. A low rate of apoptosis in irradiated CD4^+^ lymphocytes might enhance the production of cytokines, attract inflammatory immune cells to the irradiated tissue, and drive polarisation towards a pro-fibrotic response^17,19,30^. The CD4^+^ subtypes Th2 and Th17 are pro-fibrogenic via secreted cytokines and their effects on fibroblasts and ECM deposition (reviewed in ref. ^31^). T_reg_ cells control Th17, and the Th17/T_reg_ ratio has been associated with fibrosis, but T_reg_ cells also secrete TGF-β1 and IL-10, which stimulate and inhibit ECM deposition, respectively, and have been reported to have pro- or anti-fibrogenic effects^31^.

The CTGF minor allele (T) was protective in a recessive fashion against fibrosis and was associated with low CD4^+^ RILA, although low-RILA was associated with an increased rate of fibrosis. This implies that the direct, protective effect of the TT genotype dominates over the association of low RILA values with increased fibrosis.

CTGF plays an important role in the regulation of ECM homoeostasis. Along with TGF-β1 and downstream signaling proteins, CTGF controls the production of collagen and other ECM components^32–34^. Preclinical studies have reported that blocking CTGF may stop or, in some cases, even reverse the process of fibrosis development^35–37^. A previous study showing an association of the rs9399005 SNP with systemic sclerosis reported a higher affinity of regulatory proteins for the minor allele (T) compared to the major (C) allele and detected an alternative isoform of *CTGF* mRNA^38^. Our in vitro study on fibroblast strains with different *CTGF* genotypes showed that the T-allele influences expression levels of CTGF itself and a large number of DEGs, especially after irradiation, thus supporting the notion that the CTGF rs9399005 SNP has a regulatory function with biological consequences. Further, preliminary in-silico analysis indicated significantly higher binding affinity of transcription factors to the region containing the T allele, and binding was much more frequent on the minus strand from which CTGF is transcribed (Sami et al., in preparation). However, considering the relatively small number of fibroblast strains and potential confounding effect between the genotypes and the cell proliferation due to stochastic interactions, further studies are needed to reveal and confirm the indicated biological function of the CTGF rs9399005 SNP.

The *NBS1* gene product, nibrin, is a key component of the MRE11-RAD50-NBS1 (MRN) complex, which plays a critical role in detecting and stabilizing DNA double-strand breaks during repair. The SNP (rs1805794) is located in the exon region responsible for interaction with Sp100 nuclear antigen (SP100), near the BRCA1 C-terminal (BRCT) domain, and a nearby mutation (176:Tyr→Ala) was reported to block radiation-induced phosphorylation^39^. Previously, no association was found with acute normal-tissue reactions^40^ nor with telangiectasia at 4.3 years mean follow-up after radiotherapy^41^.

Although the risk of hypertension is known to increase with overweight, multivariate analysis showed that the effects of BMI and hypertension were independent. The data-driven BMI cutoff defined by the PA algorithm (BMI = 24.4 kg/m^2^) is close to the WHO threshold for overweight in females (BMI = 25 kg/m^2^), indicating that the PA cutpoint was biologically meaningful. A high BMI has been associated with increased risk of liver fibrosis, which may be related to an enhanced inflammatory state associated with visceral fat^42,43^. Large breast size and hypertension were associated with fibrosis after hypofractionated radiotherapy^44^. Furthermore, hypertension is a risk factor for myocardial fibrosis^45–47^ and has been implicated in the development of liver and kidney fibrosis^48^.

In conclusion, the present study provides proof-of-concept for the subgroup hypothesis that certain risk factors for breast fibrosis after radiotherapy are conditional on the presence of other risk factors. Thus, a significant association within a subgroup may be obscured in the classical approach by statistical ‘noise’ from complementary subgroups. Robust ML modelling confirmed the subgroup hypothesis with five-fold variation in risk and identified a smaller subgroup of high-risk patients, thus enabling biologically-aware risk stratification. Using just five features, risk stratification by this approach improved prediction over classical regression models and performed on par with more complicated models using dozens or hundreds of SNPs or other risk factors. If externally validated in an independent cohort, these advances could potentially enable more precise risk assessment of late toxicity for patients receiving breast cancer radiotherapy. The approach may even have broader applicability in personalised medicine, especially if additional interacting risk factors are identified in the future.

## Methods

### Patients, treatments, and endpoints

All patients belonged to the German ISE cohort on radiation sensitivity, recruited prospectively during 1998-2001 from the Rhine-Neckar region in Germany^49^. The median age of the patients was 59 years at the time of breast cancer diagnosis, and the patients had been treated with breast-conserving surgery followed by RT, without chemotherapy. Adjuvant RT was given to the whole breast (50.0 Gy in 2.0 Gy/fraction or 50.4 Gy in 1.8 Gy/fraction) followed by a tumour-bed boost of 6 to 16 Gy (median: 10 Gy, 2.0 Gy/fx; *n* = 83), or with 56.0 Gy given in 2.0 Gy fractions without a boost. Ethics approval for the original follow-up was obtained from the ethics committee of the Medical Faculty, Heidelberg University (approval number: 062/2002), with an amendment for the late follow-up, including RILA (approved on 17.12.2010). Fibrosis and telangiectasia were scored according to the LENT-SOMA scoring system, and blood samples were taken with written informed consent according to the Declaration of Helsinki principle^19^. Patient scores were dichotomised with grades 0–1 considered fibrosis/ telangiectasia-negative, and grades 2-3 considered fibrosis/telangiectasia-positive. At median follow-up of 11.6 years (10.3–12.8 years), positive rates were 30.6% for fibrosis and 9.5% for telangiectasia in 252 patients with RILA data, and DNA was available from 238 patients. RILA was performed at the time of the late follow-up, as previously described^19^.

### Candidate SNP selection, genotyping, and power analysis

Based on a pilot screening, three candidate SNPs, rs9399005 in the *CTGF* gene, rs1805794 in *NBS1*, and rs373759 in *ATM*, were selected for genotyping by allele discrimination with real-time quantitative polymerase chain reaction. DNA was extracted from whole blood or buffy coat using FlexiGene DNA kits (QIAGEN, Hilden, Germany), as previously described^50^. Genotyping was performed by allelic discrimination using TaqPath™ qPCR Master Mix (Applied Biosystems, Massachusetts, US) with appropriate primer solutions and analyzed on a StepOnePlus™ machine (Applied Biosystems) according to the manufacturer’s instructions. Primer ordering IDs were: C_30290305_20 (rs9399005), C_26470398_30 (rs1805794), C_1039777_20 (rs373759). Validation of ambiguous genotypes, and genotyping of the rs9399005 SNP for selecting fibroblasts from the GENEPI cohort was performed by Sanger sequencing (primers included in Supplementary Table S3).

A power analysis for additive genetic models was performed in R, using the Power Genetics library^51^ for 238 samples for a power of 0.8 at α = 0.05. Minor allele frequencies (MAF) for Utah residents with Northern and Western European (CEU) ancestry in the 1000 Genomes Project dataset were used^52^.

### Cell culture and irradiation

Tests of phenotypic effects of the rs9399005 (*CTGF*) genotypes were performed with early-passage skin fibroblasts from a local biobank established with written informed consent as part of the European GENEPI project by ESTRO and EURATOM^53,54^ (Medical Ethics Commission II, Universitätsmedizin Mannheim, approval number 184/04). Early-passage fibroblast cultures were grown in AmnioMAX^TM^ C-100 medium (Thermo Fisher, Darmstadt, Germany), supplemented with 7.5% AmnioMAX^TM^ C-100 supplement, 7.5% foetal bovine serum (HyClone, Fisher Scientific GmbH, Schwerte, Germany), 2 mM glutamin, and penicillin/streptomycin. For clonogenic cell survival, the colony formation assay (CFA) was performed as described previously^55,56^. Cell irradiation was performed with 6 MV X-rays from a clinical therapy machine, and dosimetry was part of routine quality checks.

### Gene expression analysis

Total cellular RNA was extracted on day 0 from non-irradiated fibroblasts and on day 2 from irradiated and non-irradiated fibroblasts using Qiagen RNeasy Mini Kit (Qiagen, Hilden, Germany), according to the manufacturer’s instructions. RNA sequencing was performed by a commercial company, and raw data were obtained as FASTQ files. Raw gene read counts from the RNA-seq data were calculated with the Kallisto (pseudoalignment) program. Gene normalisation (voom function) and differential gene expression (DGE) analysis were performed with the limma package in R. Nominal p-values were calculated using the moderated t-test and were adjusted for multiple testing by the Benjamini-Hochberg (BH) method.

### Feature independence and subgroup analysis

Test of collinearity between the features was performed by the Variance Inflation Factor (VIF) method in Python using the “variance_inflation_factor” function from the “statsmodels” library.

### Interaction analysis

To formally test the effect of interactions between the features, a multivariable logistic regression model was created. The model included the interactions previously suggested by PA and tested manually (RILA:CTGF, RILA:NBS1, RILA:BMI, RILA:HTN, BMI:NBS1, RILA:NBS1:BMI). The full model formula is following:

$$\begin{array}{l}logit\{P(Yi=1)\}\,=\beta 0+\beta {1}_{RILA}+\beta {2}_{BMI}+\beta {3}_{HTN}\\ +\beta {4}_{CTGF}+\beta {5}_{NBS1}+\beta {6}_{RILA\times BMI}\\ +\beta {7}_{(RILA\times HTN)}+\beta {8}_{(RILA\times CTGF)}\\ +\beta {9}_{(RILA\times NBS1)}+\beta {10}_{(NBS1\times BMI)}\\ +\beta {11}_{(RILA\times NBS1\times BMI)}\\ \,\end{array}$$

Robust (HC3) sandwich standard errors were used for coefficient inference, given the relatively small sample size. Odds ratios and nominal p-values were calculated by using the Wald test. P-values were adjusted for multiple testing with the Benjamini-Hochberg (BH) method.

For visualization, interaction plots and *p*-values were created based on independent per-group logistic regression. P-values from average marginal effect analysis were calculated by a joint multivariable model for the corresponding simple slope (marginal effect) of the x-variable at that subgroup’s moderator values.

### Machine learning (ML) models

The development of RF and SVM classifiers is presented schematically in Supplementary Fig. S9. The RF algorithm is based on creating a large number of decision trees (DTs) on different training subsets of the whole data set and making the final prediction by majority vote of the DTs. The SVM model was developed with a Radial Basis Function kernel, and classification based on the differences of inner outliers, or samples that are most similar to the opposite class (i.e., fibrosis negative samples that are similar to fibrosis positive samples vs fibrosis positive samples that are similar to fibrosis negative samples). The whole process was performed by using the Python programming language and the Scikit-Learn framework for Python^57^.

The hyperparameter values used in the models were defined by applying the GridSearchCV function from the Scikit-Learn library (Python) and 10-fold stratified CV with applied standard scaler in a pipeline, using AUC as referent selection metric. The parameter values were as follows:

RF model: Max depth (of the trees) = 5, Max leaf nodes = 30, Max samples used per tree = 0.9 (of the whole cohort), Min samples per leaf = 3, Min samples per split =10, Number of estimators (trees) = 500. SVM model: Regularization parameter (C) = 20, Gamma = 0.05, Class weights = balanced (automatically balanced according to the ratio between the classes), Kernel = Radial Basis Function (RBF). Non-indicated parameters kept default values (according to Scikit-Learn).

Because of the intermediate sample size of the cohort, Leave-One-Out Cross-Validation (LOOCV) was used and implemented in all downstream analysis and evaluation methods. This means that the prediction for each sample was made based on the model trained on the rest of the cohort, as presented in Fig. S9.

The stability of the final models was verified by additional stratified 10-fold cross-validation repeated ten times. To compute the error rates with stratified 10-fold cross-validation, the whole dataset was randomly split into 10 subgroups (folds), preserving the ratio of fibrosis-positive and fibrosis-negative samples (~30% of fibrosis-positive samples) in each subgroup. Each subgroup was then used as a test set, while training the models on the other nine subgroups, calculating error rates of predicted fibrosis status in the test and training sets. Thus, ten pairs of error rates were obtained from ten train-test pairs. The whole process was repeated 10 times, making ten new subgroups each time, which created 100 train-test pairs in total. Error rates and standard deviations for training and test datasets were calculated from all 100 pairs of error rates.

The RF and SVM models were compared with Logistic Regression (LR: a linear model) and other commonly used ML models: Extreme Gradient Boosting (XGB: an ensemble model), K-Nearest Neighbour (KNN), and Naïve Bayes Classifier (NBC). Model performance was evaluated by precision (positive and negative predictive values), accuracy, Receiver Operating Characteristic (ROC) area under the curve (AUC), and Cohen’s kappa coefficient. All models were created using the Scikit-Learn library, except XGB, which was developed from the XGBoost library^58^. The LOOCV method was applied to all models.

### Tests for feature and model evaluation

The characteristics of the models were evaluated by the cumulative accuracy profile (CAP), with predicted probabilities of positive outcomes (based on the predictions from LOOCV) as a function of the proportion of the dataset. The accuracy ratios (AR) were determined as the ratio of the areas under the curve of the test model vs. the perfect model.

The influence of each feature on the models was evaluated by the Permutation Feature Importance test, which scores the average decrease of model accuracy when values of each feature are shuffled multiple times (LOOCV performed for each permutation). This implies shuffling values in each feature a number of times, every time repeating the whole model construction process and measuring the accuracy of the model. Finally, the importance of each feature is presented as an average decrease in model accuracy after shuffling the values for that feature.

The statistical significance of the final models was evaluated by a permutation test^59^. The dataset was shuffled a number of times (i.e., 1000 times), each time making predictions based on the developed models (LOOCV applied) and calculating accuracies. This way, the distribution of accuracies for randomized datasets was created. The p-value is calculated as the fraction of randomized datasets where the accuracy is higher than in the original dataset.

Data manipulation and mathematical calculations were performed with NumPy^60^ and Pandas^61^ Python libraries. The related figures were created in the Matplotlib library for Python^62^.

### Statistical analysis

Basic statistical analyses were performed with JMP statistical software (SAS, Böblingen, Germany). Associations between categorical variables were assessed using contingency table tests. For 2 × 2 tables, Fisher’s exact test (two-tailed) was used; for tables larger than 2 × 2, Pearson’s χ² test was applied (as implemented in JMP). For non-parametric testing of continuous parameters between two groups, the Mann-Whitney U test, and for comparing more than two groups, the Kruskal-Wallis test were used. Potential subgroups were identified by partition analysis (PA), which creates a decision tree (DT) by a number of consecutive dichotomous splits. The risks of fibrosis in complementary subgroups were compared by contingency analysis using Fisher’s exact test. The effect of BMI on the RILA-fibrosis relationship according to HTN status was determined using the ‘Fit model’ linear model function.

## Supplementary information

Manuscript_ISE Subgroup hypothesis_suppl mat - rv1 amended

Supplementary Data 1_DEG tables

## Data Availability

Due to the sensitive nature of the clinical data and data protection regulations, the clinical data are not publicly available. Upon reasonable request, a reduced data set may be available subject to a data access agreement. The differentially expressed genes are available in Supplementary Data 1, and the raw data are deposited in the GEO repository as private files (accession number: GSE325569). They will be available to reviewers and editors, or upon reasonable request, and will be made publicly available after full publication of the gene expression and pathway analysis.

## Code availability

The machine learning models were developed using publicly available, standard Python libraries and functions (sklearn, numpy, pandas, etc.). Gene expression analysis was performed in R. No custom functions were used in developing the models.
