# Supplementary material for: Novel method for risk stratification of radiation-induced breast fibrosis: subgroup hypothesis verified by machine learning
Source: NPJ Breast Cancer. 2026 Jun 12;12:84. doi: 10.1038/s41523-026-00980-7 (PMC13279946; doi:10.1038/s41523-026-00980-7)
Supplement: Supplementary file 1 — Manuscript_ISE Subgroup hypothesis_suppl mat - rv1 amended [file 41523_2026_980_MOESM1_ESM.pdf]

Supplementary Material.

Sami *et al.*: **Novel method for risk stratification of radiation-induced breast fibrosis: subgroup hypothesis verified by machine learning**

Supplementary results.

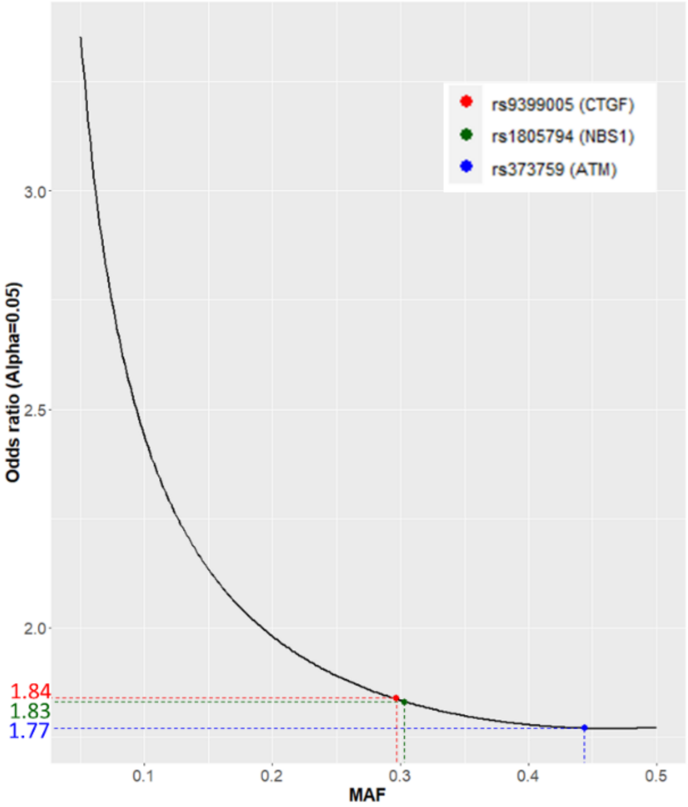

Supplementary Figure S1. Power analysis showing the Odds Ratios (OR) that should be detectable with a power of 80% at α=0.05 with 238 samples as function of the minor allele frequency (MAF), according to the additive genetic model.

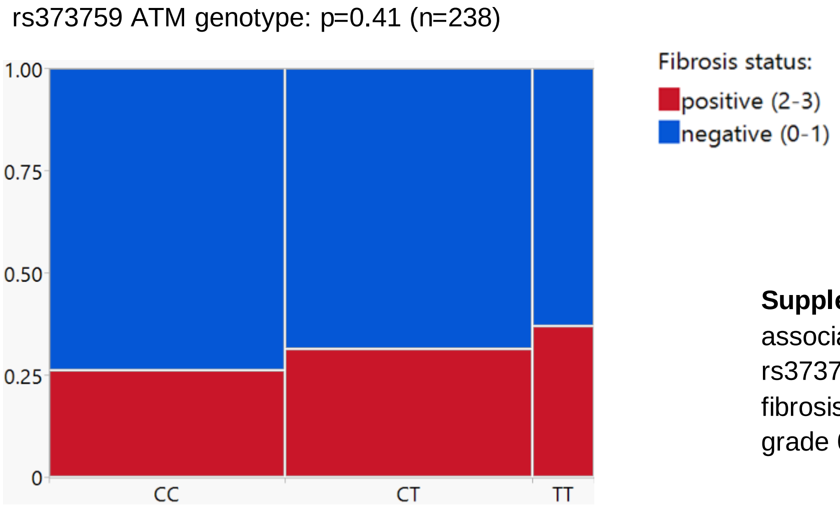

Supplementary Figure S2. No association between *ATM* SNP rs373759 and radiation-induced fibrosis grade 2-3 (fib-pos) versus grade 0-1 (fib-neg).

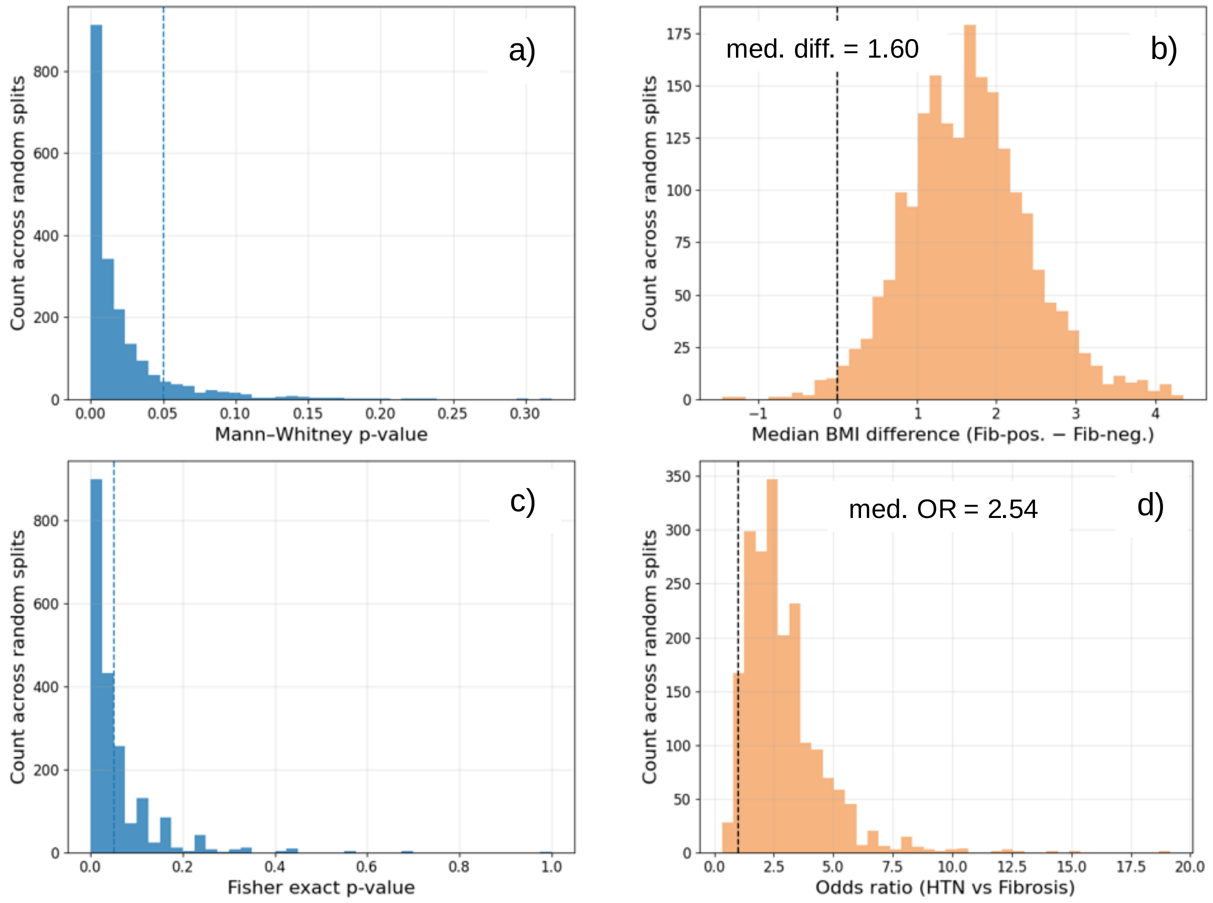

Supplementary Figure S3. Distribution of statistical estimates across 2000 train/test splits (70/30) keeping the same fibrosis rate in each subset. a) Distribution of the p-values (Mann-Whitney U test) testing the association between BMI and fibrosis. b) Distribution of differences in BMI between fibrosis positive and fibrosis negative patients. c) Distribution of the p-values (Fisher's exact test) testing the association between HTN and fibrosis. d) Distribution of odds ratios showing risk of fibrosis in HTN positive vs HTN negative patients.

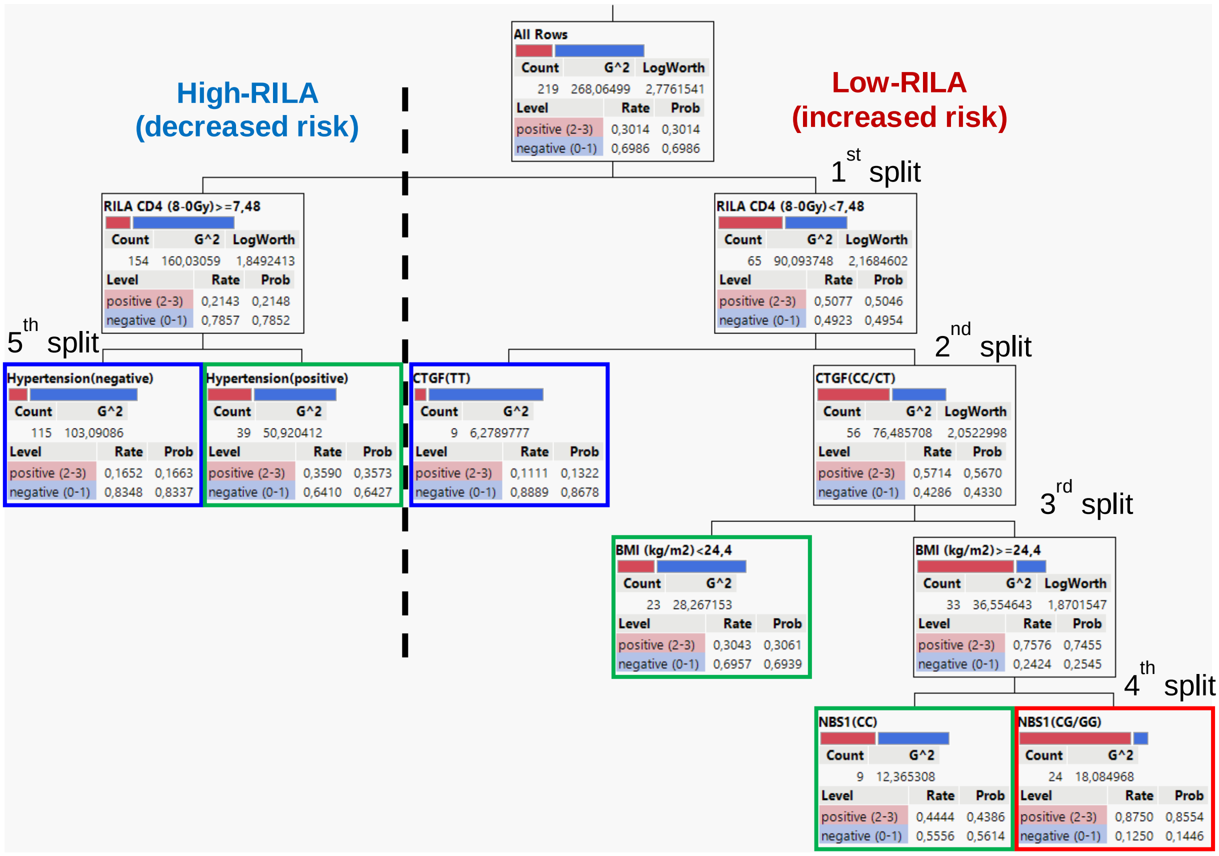

Supplementary Figure S4: Decision Tree (DT) for predictive partition analysis (PA). The first split was according to a $CD4^+$ RILA with lower values (< 7.48%; n=65) associated with increased risk (50.1% fibrosis) and higher values (≥ 7.48%; n=154) with relative resistance (21.4% fibrosis). The second split separated the low-RILA group based on rs9399005 (*CTGF*) genotype in a high-risk group (CC/CT; n = 56) with 57.1% fibrosis and a highly resistant subgroup (TT; n = 9) with 11.1% fibrosis in spite of being in the low-RILA group associated with higher risk. The third split separated the high-risk group according to BMI where 75.8% of the patients with BMI ≥ 24.4 kg/m^2 (n=33) developed fibrosis whereas the rate was only 30.4% among patients with BMI < 24.4 kg/m^2 (n=23). The fourth split divided the high-risk group based on the rs1805794 (*NBS1*) genotype into a very high risk group of heterozygous and minor homozygous genotypes (CG/GG) with 87% fibrosis (n=24) against 44% risk in major homozygotes (CC; n=9). Notably, this also indicated that the CC genotype was under-represented in the low-RILA/ BMI(high) group. Finally, the fifth split divided the high-RILA group based on hypertension status at diagnosis, where 35% of patients with hypertension (n=39) developed fibrosis but only 16.5% of patients with normal blood pressure. The feature contributions (from JMP Predictive Modeling/PA) were: $CD4^+$ RILA (36.6%), BMI (23.8%), Hypertension status (17.3%), *CTGF* minor homozygote TT (14.9%), and *NBS1* major homozygote CC (12.4%). The six final subgroups could be grouped into three risk groups, low-risk (blue bordered leaves), medium-risk (green border leaves), and high-risk (red bordered leaf). The black broken line separates risk groups according to the RILA $CD4^+$ subgroups.

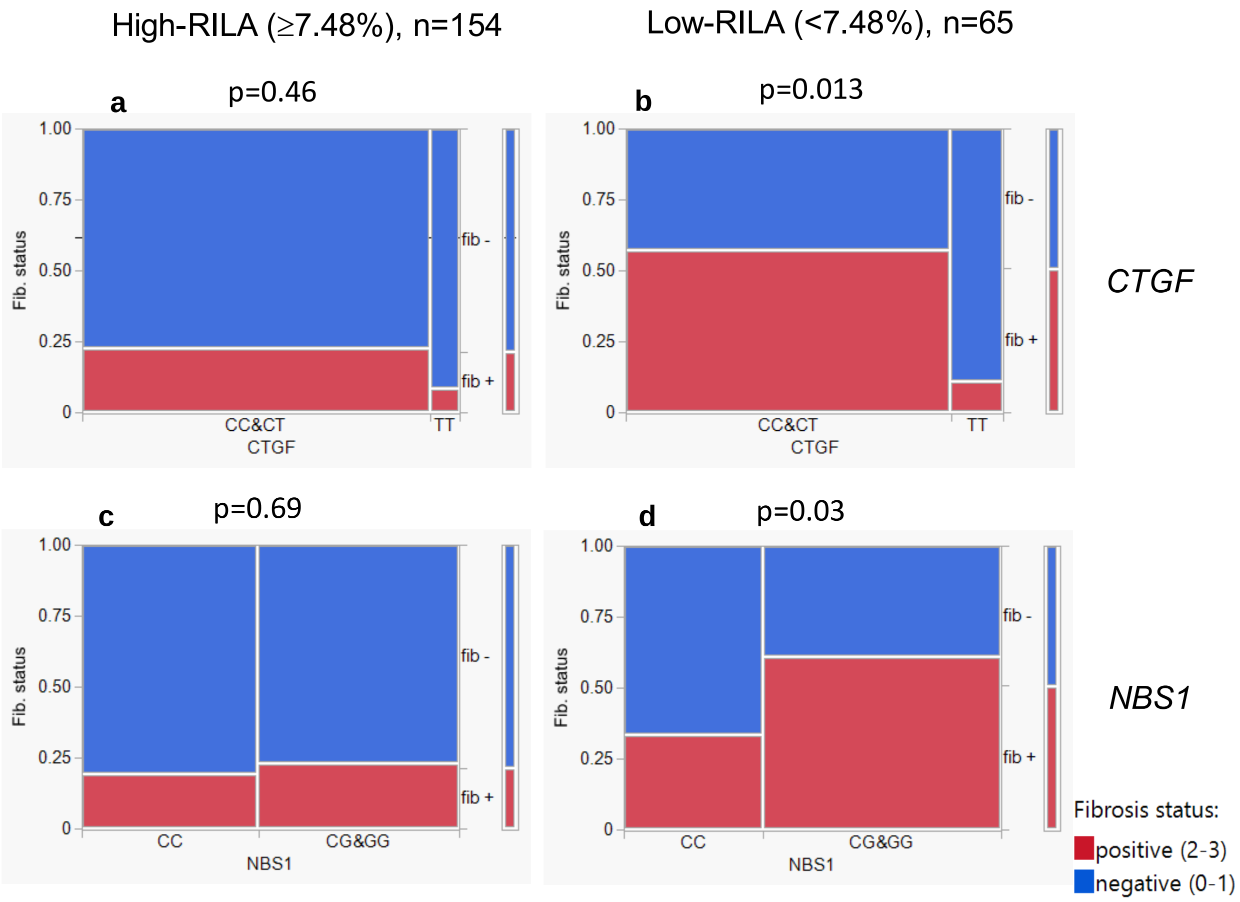

Supplementary Figure S5a-d. Test of the subgroup hypothesis. Mosaic plots of contingency analysis for *CTGF* and *NBS1* SNPs by RILA subgroup. High-RILA: ≥7.48%, low-RILA: <7.48%. Fisher's exact test (2-sided). The *CTGF* TT genotype was significant in the smaller low-RILA subgroup, showing a lower p-value than in the whole group of 219 patients (p=0.013 versus p=0.043) while it was not significant in the complementary and larger high-RILA subgroup (p=0.46). Similarly, the *NBS1* CG&GG genotype was more significant (p=0.032) in the low-RILA subgroup than in the whole group (p=0.053) while it was not significant in the complementary high-RILA subgroup (p=0.69).

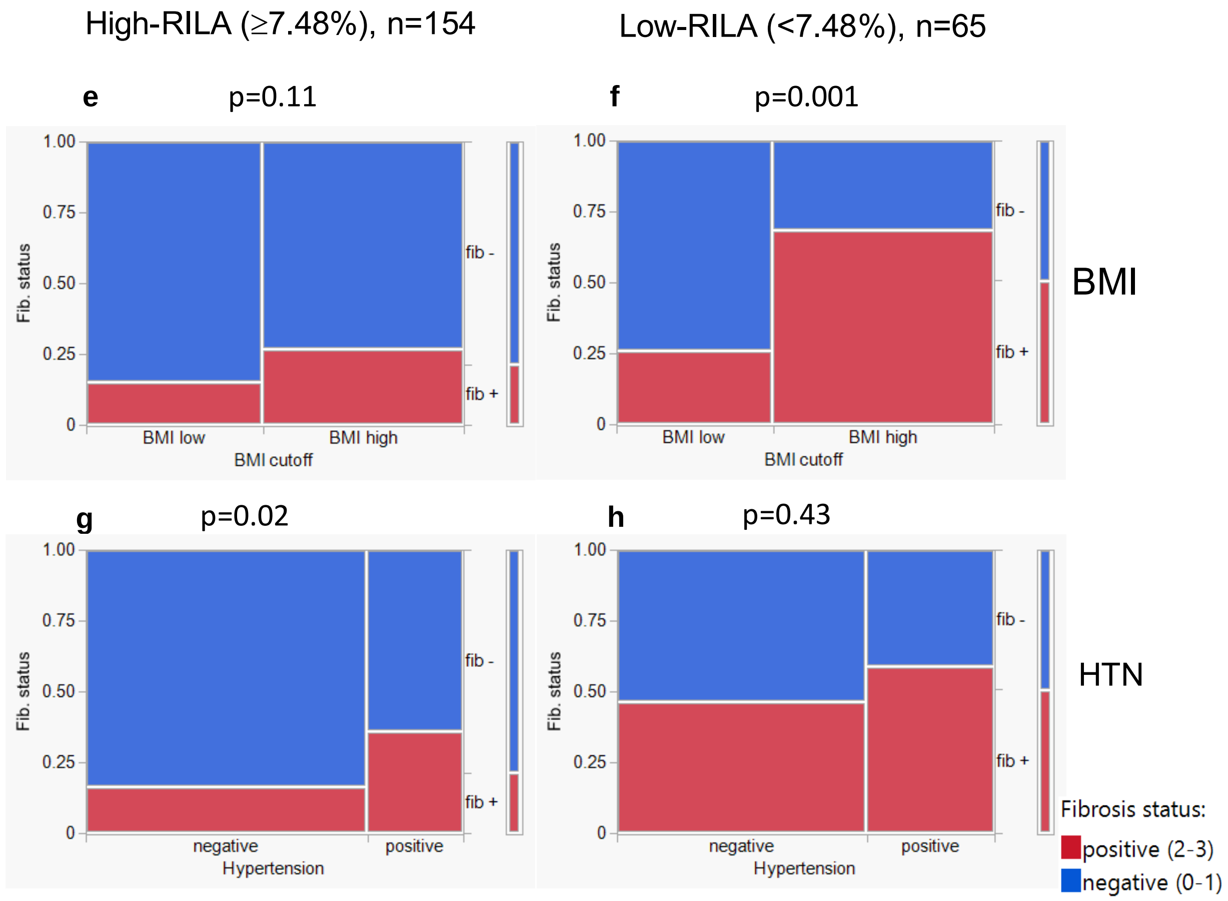

Supplementary Figure S5e-h. Test of the subgroup hypothesis. Mosaic plots of contingency analysis for BMI and hypertension (HTN) by RILA subgroup. High-RILA: ≥7.48%, low-RILA: <7.48%. BMI(high): ≥24.4 kg/m^2, BMI(low) <24.4 kg/m^2. Fisher's exact test (2-sided). BMI (≥24.4 kg/m^2) was highly significant in the low-RILA subgroup (p=0.001) and in the whole group (p=0.0006) but also showed a non-significant trend (p=0.11) in the high-RILA subgroup. By contrast, hypertension was significant in the high-RILA subgroup (p=0.02) and in the whole group (p=0.008) but not in the low-RILA subgroup (p=0.43).

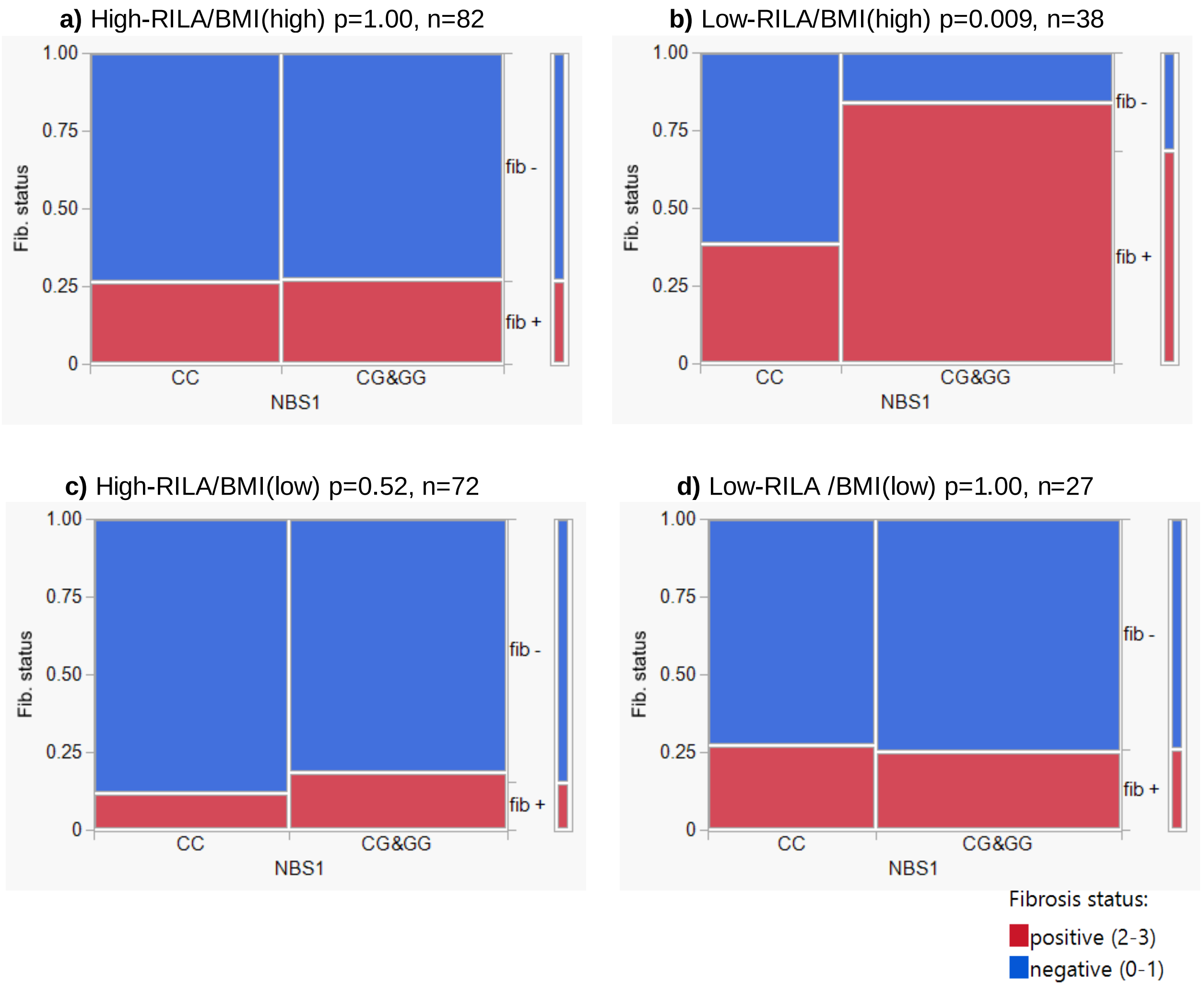

Supplementary Figure S6a-d. Subdivision of the RILA subgroups according to BMI. Mosaic plots of contingency analysis for the NBS1 SNP by subgroups defined by combinations of RILA & BMI. High-RILA: ≥7.48%, low-RILA: <7.48%. BMI(high): ≥24.4 kg/m^2, BMI(low): <24.4 kg/m^2. Fisher's exact test (2-sided). Combining RILA subgroups with BMI status showed that the *NBS1* SNP was only significant in the sub-subgroup low-RILA/BMI(high) (p=0.009; b) but not in any of the other three combinations (p=0.52-1.00; a, c-d).

a) rs9399005 *CTGF* (n=223). b) rs1805791 *NBS1* (n=223). rs373759 *ATM* (n=223).

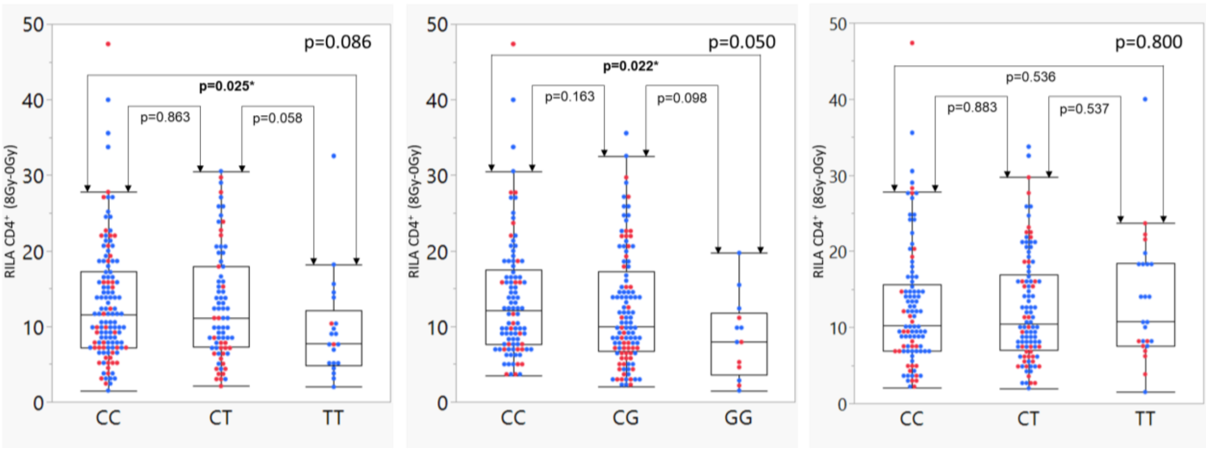

d) CDF plot for *CTGF* SNP. e) CDF plot for *NBS1* SNP.

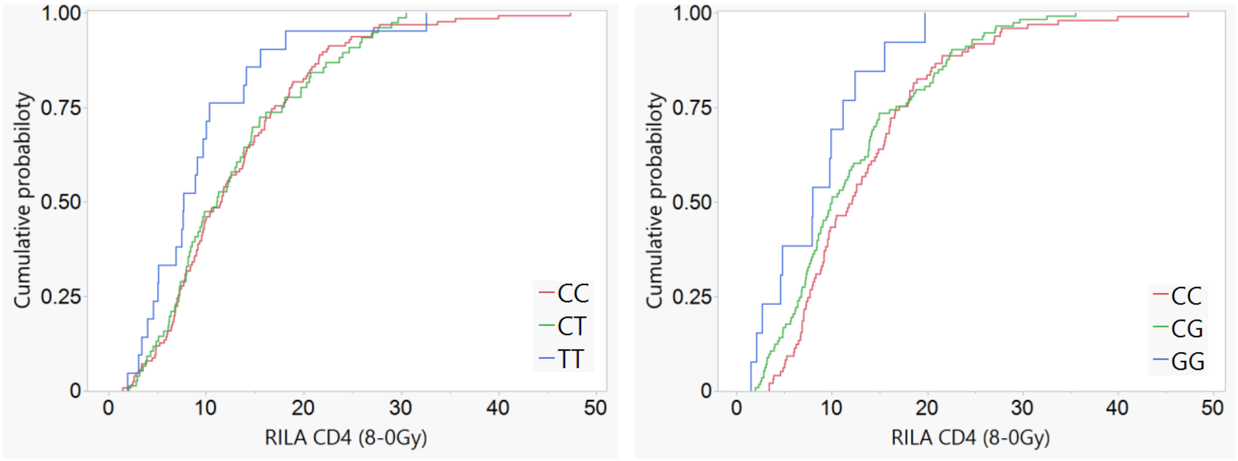

Supplementary Figure S7a-e. Interactions of SNP genotypes with CD4$^+$ RILA showed significant associations for a) *CTGF and* b) *NBS1* but not c) *ATM*. Cumulative Distribution Function (CDF) plots of RILA CD4$^+$ values for d) rs9399005 (*CTGF*) showed lower RILA values for the minor homozygote (TT) genotype and e) a dependence on the number of rs1805794 (*NBS1)* minor alleles (G) below approximately 16-17% but not at higher values.

f) RILA vs BMI g) RILA vs HTN

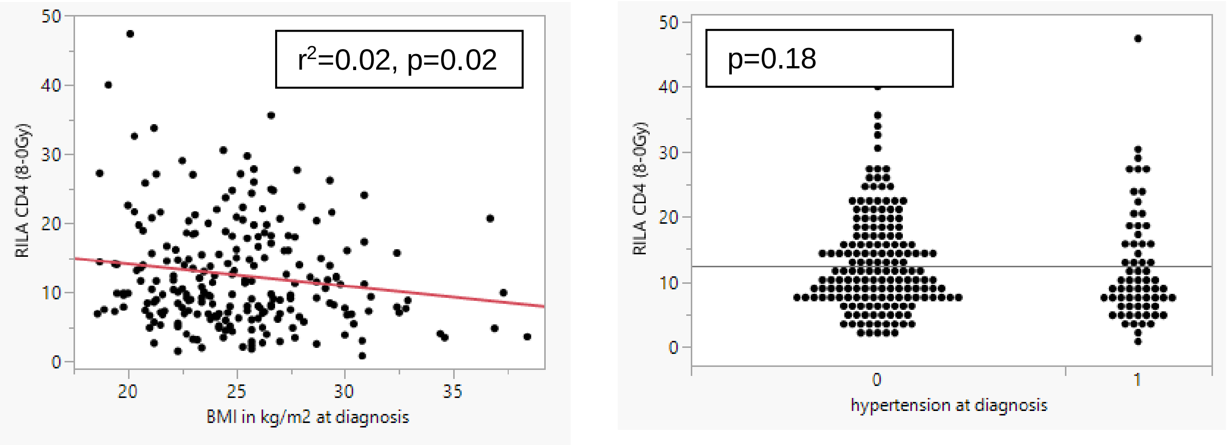

Supplementary Figure S7f,g. Interactions of clinical features body mass index (BMI) and hypertension (HTN) with CD4^{+} RILA. f) The association between BMI and RILA was very weak (r^2= 0.02, p=0.02, n=234, lin. regr.); g) no significant association between HTN and RILA was observed (p=0.18, n=234, Mann-Whitney).

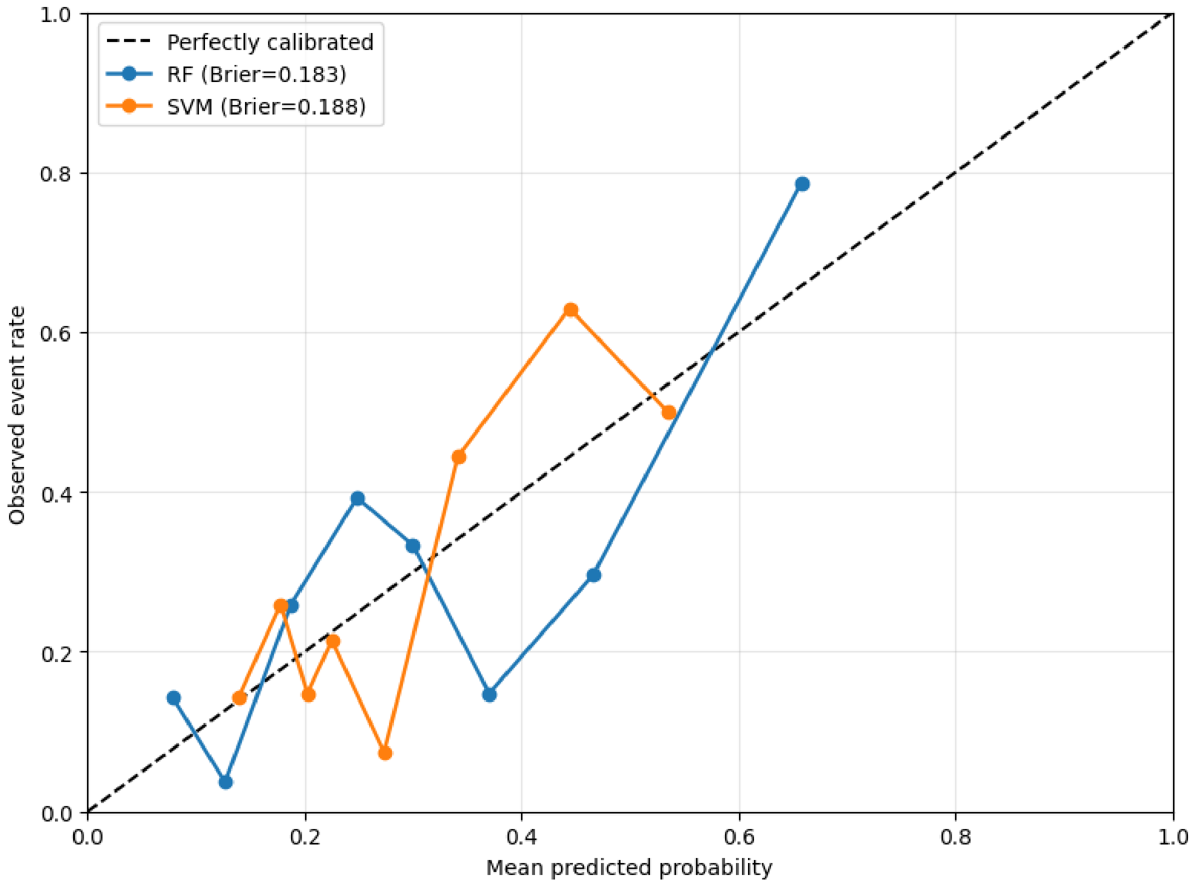

Supplementary Figure S8. Brier scores and the calibrations curve for AUC_ROC and CAP analysis computed based on the predicted probability values (not binary predictions) of the RF and SVM models.

Supplementary Table S1. Statistical tests of logistic regressions shown in Figure 3a-c.

a) Test results for logistic regression of each individual curve

Feature	Value	Subset	n	slope logit per unit	SE	p_wald	OR per_unit	OR_95% CI_low	OR_95% CI_high
CTGF (Fig. 3a)	CC&CT	BMI < 24.4	89	0.0174	0.0346	0.6143	1.0176	0.9509	1.0889
CTGF (Fig. 3a)	CC&CT	BMI ≥ 24.4	109	-0.0661	0.0313	**0.0347**	0.9361	0.8804	0.9953
CTGF (Fig. 3a)	TT	BMI < 24.4	10	-0.0010	0.0424	0.9804	0.9990	0.9193	1.0855
CTGF (Fig. 3a)	TT	BMI ≥ 24.4	11	-0.0701	0.0751	0.3501	0.9323	0.8048	1.0800
NBS1 (Fig. 3b)	CG&GG	BMI < 24.4	54	0.0411	0.0417	0.3242	1.0420	0.9602	1.1308
NBS1 (Fig. 3b)	CG&GG	BMI ≥ 24.4	69	-0.0934	0.0428	**0.0291**	0.9108	0.8376	0.9905
NBS1 (Fig. 3b)	CC	BMI < 24.4	45	0.0031	0.0647	0.9622	1.0031	0.8835	1.1388
NBS1 (Fig. 3b)	CC	BMI ≥ 24.4	51	0.0209	0.0468	0.6554	1.0211	0.9316	1.1192
HTN (Fig. 3c)	No HTN	BMI < 24.4	78	-0.0112	0.0419	0.7895	0.9889	0.9110	1.0735
HTN (Fig. 3c)	No HTN	BMI ≥ 24.4	80	-0.1124	0.0465	**0.0156**	0.8937	0.8160	0.9789
HTN (Fig. 3c)	HTN+	BMI < 24.4	21	0.0457	0.0376	0.2250	1.0467	0.9723	1.1268
HTN (Fig. 3c)	HTN+	BMI ≥ 24.4	40	0.0305	0.0409	0.4558	1.0310	0.9516	1.1170

b) Test results for average marginal effects analysis by joint multivariable model.

Feature	Value	Subset	n	slope logit per unit	SE	p_wald	OR per_unit	OR_95% CI_low	OR_95% CI_high
CTGF (Fig. 3a)	CC&CT	BMI < 24.4	89	-0.0222	0.0310	0.4736	0.9781	0.9205	1.0392
CTGF (Fig. 3a)	CC&CT	BMI ≥ 24.4	109	-0.0736	0.0342	**0.0316**	0.9291	0.8688	0.9935
CTGF (Fig. 3a)	TT	BMI < 24.4	10	-0.0225	0.1115	0.8404	0.9778	0.7859	1.2166
CTGF (Fig. 3a)	TT	BMI ≥ 24.4	11	0.0120	0.1157	0.9176	1.0120	0.8067	1.2697
NBS1 (Fig. 3b)	CG&GG	BMI < 24.4	54	0.0036	0.0380	0.9249	1.0036	0.9315	1.0813
NBS1 (Fig. 3b)	CG&GG	BMI ≥ 24.4	69	-0.1331	0.0466	**0.0042**	0.8754	0.7990	0.9590
NBS1 (Fig. 3b)	CC	BMI < 24.4	45	-0.0532	0.0482	0.2700	0.9482	0.8627	1.0422
NBS1 (Fig. 3b)	CC	BMI ≥ 24.4	51	0.0254	0.0512	0.6191	1.0258	0.9279	1.1340
HTN (Fig. 3c)	No HTN	BMI < 24.4	78	-0.0504	0.0379	0.1837	0.9509	0.8828	1.0242
HTN (Fig. 3c)	No HTN	BMI ≥ 24.4	80	-0.1084	0.0391	**0.0055**	0.8973	0.8311	0.9687
HTN (Fig. 3c)	HTN+	BMI < 24.4	21	0.0824	0.0365	**0.0239**	1.0859	1.0109	1.1664
HTN (Fig. 3c)	HTN+	BMI ≥ 24.4	40	0.0196	0.0501	0.6958	1.0198	0.9244	1.1250

Supplementary Table S2a. Comparison of ML model performances based on the five features ($CD4^+$ RILA, CTGF and NBS1 SNPs, BMI, and Hypertension). LR: logistic regression, XGB: eXtreme Gradient Boosting, KNN: K-nearest neigbour, NBC: Naïve Bayes Classifier. The RF model was more precise in predicting fibrosis positive samples (PPV of 78% compared 56% for SVM), while SVM was more precise in predicting fibrosis negative patients (NPV of 83% compared to 78% for RF).

Performance indicator	RF	SVM	LR	XGB	KNN	NBC
NPV	0.78	0.83	0.80	0.77	0.78	0.77
PPV	0.78	0.56	0.43	0.62	0.54	0.46
Recall Fib-	0.95	0.79	0.65	0.90	0.83	0.76
Recall Fib+	0.38	0.62	0.62	0.39	0.45	0.47
Accuracy	78.1%	74.0%	63.9%	74.4%	71.7%	67.6%
Kappa coefficient	0.39	0.40	0.24	0.32	0.30	0.23
AUC (ROC)	66.7%	70.6%	63.4%	64.5%	64.2%	61.7%

Supplementary Table S2b. Confusion matrices of actual versus predicted outcomes (true and false predictions) for the RF and SVM models. The exact numbers (and percentages) of correct and incorrect predictions for fibrosis positive and negative samples are presented. For the RF model, true negative predictions represented 67% and false negatives 18.8% of predictions while true positives are 11.4% and false positives 3.2% of all predictions. In the SVM model, true negative predictions made up 55% and false negatives 11.4% of the predictions while true positives were 18.7% of predictions and false positives 14.6% of all predictions. Thus 78% (67+11.4%) and 74% (55+18.7%) of all patients were predicted correctly by RF and SVM, respectively.

	**Random forest**		**Support Vector Machine**	
	predicted negative	*predicted positive*	*predicted negative*	*predicted positive*
Actual negative	146 (67%)	7 (3.2%)	121 (55%)	32 (14.6%)
Actual positive	41 (18.8%)	25 (11.4%)	25 (11.4%)	41 (18.7%)

Supplementary Table S3. Primers used in Sanger sequencing:

	**rs9399005 (CTGF):**	**rs1805794 (NBS1):**
Outer forward PCR	5'-TATTTGGGGAGGTGGGGTGCTGCT	5’-AGGCCTTGGAAGTCCCCTTTGG
Outer reverse PCR	5'-CCCCAGTGACAGCTAGGATGTGCA	5’-TGTTGGCATGGACCTAGCTCACAA
Inner forward PCR	5'-AAGACTTGGGCAAATGGAGG	5’-AGGGAAGGATGGCAGAGAAA
Inner reverse PCR	5'-AGTATCACAGACTTGCAGGCA	5’-GATGTTGAGGACTGCTGGAA
Sanger sequencing	5'-AAGACTTGGGCAAATGGAGG	5’-AGGGAAGGATGGCAGAGAAA

a)

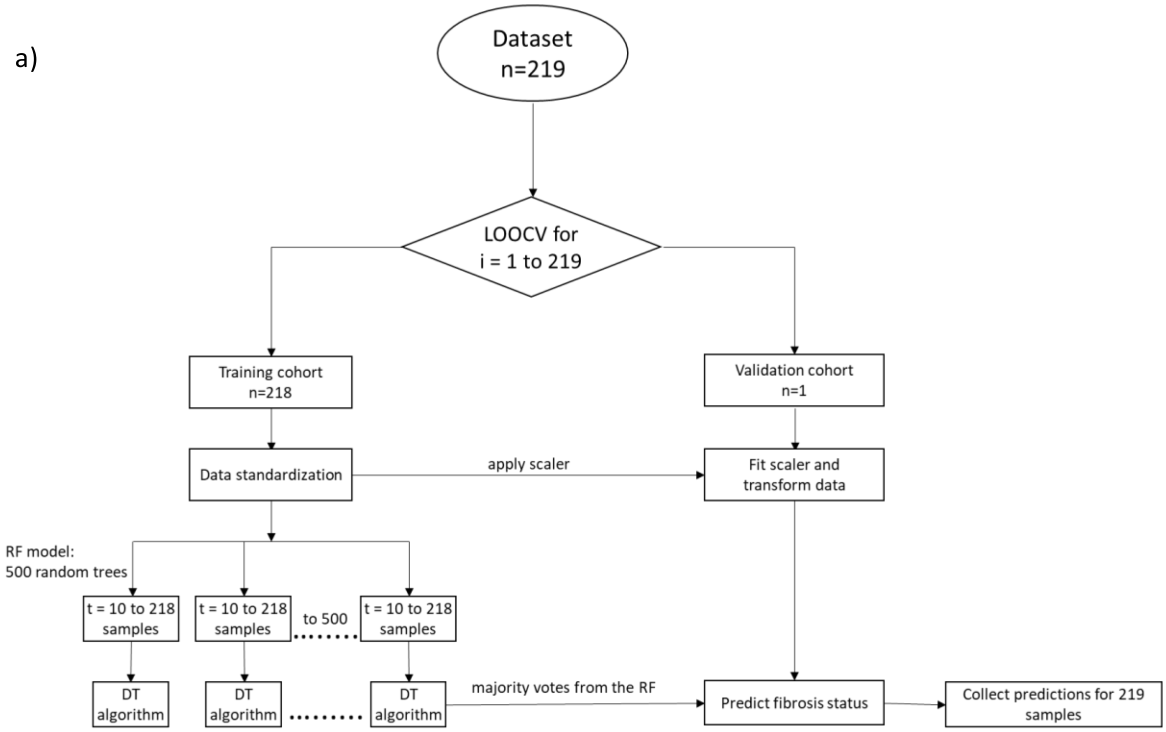

b)

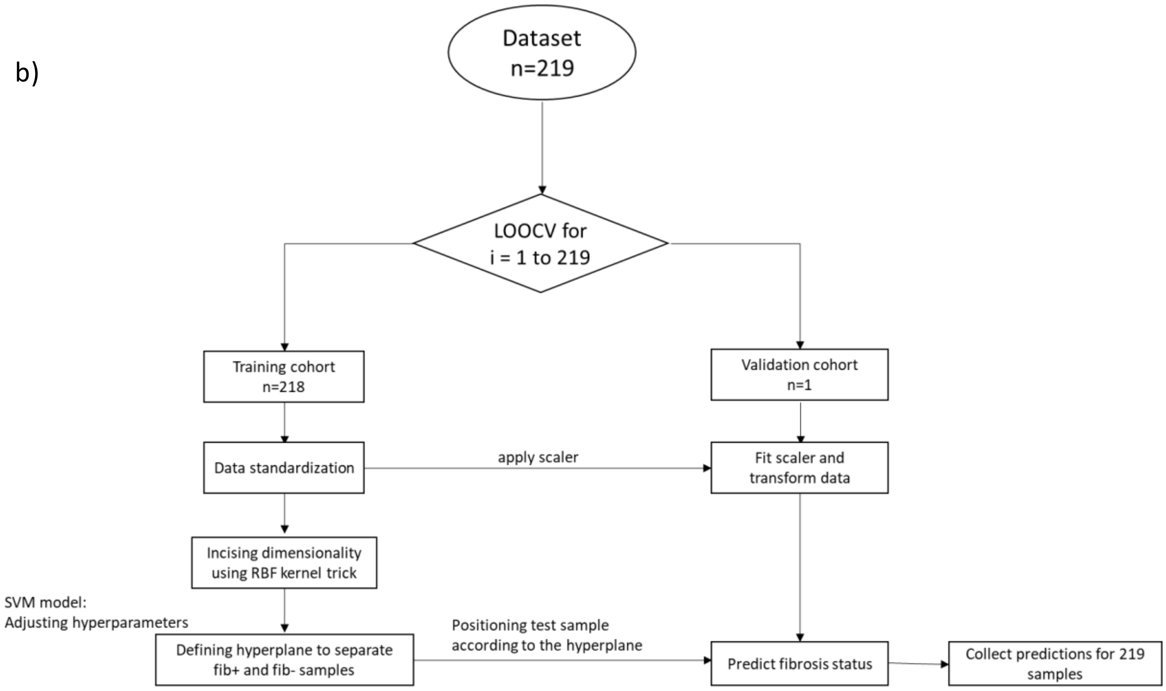

Supplementary Figure S9. Flow diagrams of the machine learning models. In both cases, leave-one-out cross-validation (LOOCV) was applied to the data set. a) RF: after splitting and scale standardizing the dataset for each LOOCV step, the RF algorithm made 500 random subgroups from the training dataset (sample sizes 10 to 218) and performed the decision tree analysis on each of them. By taking majority votes from the 500 individual trees, the test sample (n=1) was predicted as fibrosis positive or negative. b) SVM: after splitting and data scale standardization, SVM algorithm used the Radial Basis Function kernel trick to calculate the distances between the samples as they would be in a multidimensional coordinate system and considered only inner outliers to separate two classes (fibrosis positive and negative). Based on that, the model predicted the fibrosis status and classified the test sample (n=1).
